# SHIP1 modulates antimalarial immunity by bridging the crosstalk between type I IFN signaling and autophagy

**DOI:** 10.1128/mbio.03512-22

**Published:** 2023-06-27

**Authors:** Hongyu Li, Shuai Yang, Ke Zeng, Jiayin Guo, Jian Wu, Huaji Jiang, Yingchao Xie, Zhiqiang Hu, Jiansen Lu, Jianwu Yang, Xin-zhuan Su, Jun Cui, Xiao Yu

**Affiliations:** 1 Department of Immunology, School of Basic Medical Sciences, Southern Medical University, Guangzhou, Guangdong, China; 2 Guangdong Province Key Laboratory of Pharmaceutical Functional Genes, MOE Key Laboratory of Gene Function and Regulation, School of Life Sciences, Sun Yat-sen University, Guangzhou, Guangdong, China; 3 Malaria Functional Genomics Section, Laboratory of Malaria and Vector Research, National Institute of Allergy and Infectious Diseases, National Institutes of Health, Bethesda, Maryland, USA; 4 Yue Bei People's Hospital Postdoctoral Innovation Practice Base, Southern Medical University, Guangzhou, Guangdong, China; 5 Department of Joint Surgery, the Fifth Affiliated Hospital of Southern Medical University, Guangzhou, Guangdong, China; 6 Guangdong Provincial Key Lab of Single Cell Technology and Application, Southern Medical University, Guangzhou, Guangdong, China; George Washington University, Washington, DC, USA

**Keywords:** *Plasmodium*, type I interferon, autophagy, SHIP1, IRF3

## Abstract

Stringent control of the type I interferon (IFN-I) signaling is critical for host immune defense against infectious diseases, yet the molecular mechanisms that regulate this pathway remain elusive. Here, we show that Src homology 2 containing inositol phosphatase 1 (SHIP1) suppresses IFN-I signaling by promoting IRF3 degradation during malaria infection. Genetic ablation of *Ship1* in mice leads to high levels of IFN-I and confers resistance to *Plasmodium yoelii nigeriensis* (*P.y.*) N67 infection. Mechanistically, SHIP1 promotes the selective autophagic degradation of IRF3 by enhancing K63-linked ubiquitination of IRF3 at lysine 313, which serves as a recognition signal for NDP52-mediated selective autophagic degradation. In addition, SHIP1 is downregulated by IFN-I-induced miR-155-5p upon *P.y*. N67 infection and severs as a feedback loop of the signaling crosstalk. This study reveals a regulatory mechanism between IFN-I signaling and autophagy, and verifies SHIP1 can be a potential target for therapeutic intervention against malaria and other infectious diseases.

**IMPORTANCE:**

Malaria remains a serious disease affecting millions of people worldwide. Malaria parasite infection triggers tightly controlled type I interferon (IFN-I) signaling that plays a critical role in host innate immunity; however, the molecular mechanisms underlying the immune responses are still elusive. Here, we discover a host gene [Src homology 2-containing inositol phosphatase 1 (SHIP1)] that can regulate IFN-I signaling by modulating NDP52-mediated selective autophagic degradation of IRF3 and significantly affect parasitemia and resistance of *Plasmodium*-infected mice. This study identifies SHIP1 as a potential target for immunotherapies in malaria and highlights the crosstalk between IFN-I signaling and autophagy in preventing related infectious diseases. SHIP1 functions as a negative regulator during malaria infection by targeting IRF3 for autophagic degradation.

## INTRODUCTION

Malaria parasite infection activates complex and delicate immune responses, including the innate immunity which severs as the first line of host defense against invading *Plasmodium* (1, 2). Parasite DNA, RNA, protein-DNA complex, glycosylphosphatidylinositol, and hemozoin are recognized by the pattern recognition receptors of host cells, such as Toll-like receptors (TLRs), Retinoic-acid-inducible gene I (RIG-I)-like receptors, NOD-like receptors (NLRs), and nucleic acid sensors, to activate nuclear factor kappa B (NF-κB), IRF-3/7, and inflammasome signaling to produce type I interferon (IFN-I) and pro-inflammatory cytokines (3 - 5). IFN-I signaling plays an important role in inhibiting the blood stages of multiple *Plasmodium* strains at early infection (5), and its production must be tightly regulated to maintain immune balance. Recent studies have unveiled the complexity of regulation in IFN-I responses by various regulators during *Plasmodium* infections, including suppressor of cytokine signaling 1 (6), FOS-like antigen-1 (7), membrane-associated ring-CH-type finger 1 (8), and receptor transporter protein 4 (RTP4) (5). However, the complex regulatory network and the dynamics of the IFN-I pathway in response to malaria parasite infections remain unresolved (9).

SHIP1 is a member of the inositol polyphosphate-5-phosphatase (inpp5) family. SHIP1 has an N-terminal SH2 domain, an inositol phosphatase domain, and two C-terminal protein interaction domains (10) and is an important regulator of immune cell activation (11). SHIP1 dampens B cell receptor signaling through its receptor-dependent function and functions as an intrinsic brake on activation signaling (12). Moreover, substantial evidence indicates that SHIP1 controls NF-κB, mitogen-activated protein kinase, and phosphatidylinositol 3 kinases (PI3K)/serine/threonine kinase signaling via phosphatase activity. Besides, SHIP1 is also critical for hematopoietic cell development through its nonenzymatic functions (13). Given the importance of SHIP1 in regulating immune cell function and PI3K signaling, therapeutic targeting of SHIP1 has been tested in treatments of leukemia, lymphoma, and inflammatory and autoimmune disease (14). The transcription level of *inositol polyphosphate 5-phosphatase D* (*Inpp5d* or *Ship1*) has been reported to be downregulated in *Plasmodium falciparum* (*P.f*.)-infected patients (15). This suggests that it may be involved in malaria infection. However, it is unknown how SHIP1 functions in antimalarial immunity.

Autophagy is a highly conserved eukaryotic degradation process involving the cellular recycling of multiple cytoplasmic components during physiological conditions as well as in response to various stresses (16). Accumulated evidence emphasizes that autophagy is highly selective that delivers substrates such as protein aggregates and damaged or superfluous organelles to autophagosomes for lysosome degradation *via* several cargo receptors (17). Indeed, cargo receptors such as sequestosome 1 (SQSTM1/p62), CALCOCO2/NDP52, optineurin (OPTN), and neighbor of BRCA1 (NBR1), harbor both ubiquitin-binding domains and LC3-interacting regions, and direct ubiquitin-linked substrates for autophagic degradation (17, 18). Recent reports support that autophagy modulates immune responses in infectious diseases (19), but the molecular mechanism underlying the multilayer regulation of selective autophagy in antimalarial immune responses remains largely elusive (20).

In this study, we report that SHIP1 is a key negative regulator of IFN-I signaling by promoting autophagic degradation of IRF3 during malaria infection. After malaria parasite *Plasmodium yoelii nigeriensis* N67 (*P.y*. N67) infection, *Ship1*-deficient mice produce higher levels of IFN-I and have significantly lower parasitemia than wild-type (WT) mice. The association between IRF3 and cargo protein NDP52 is enhanced by SHIP1 that promotes the K63-linked ubiquitination of IRF3 at K313 and severs as a signal for NDP52-dependent selective autophagic degradation. Moreover, SHIP1 is downregulated by IFN-I-induced miR-155-5p after malaria parasite infection and functions as a feedback loop between IFN-I signaling and autophagy. This study reveals an important role of SHIP1 in mediating the crosstalk between IFN-I response and autophagy during antimalarial immune responses.

## RESULTS

### SHIP1 impairs host antimalarial immune responses

**Fig 1:**
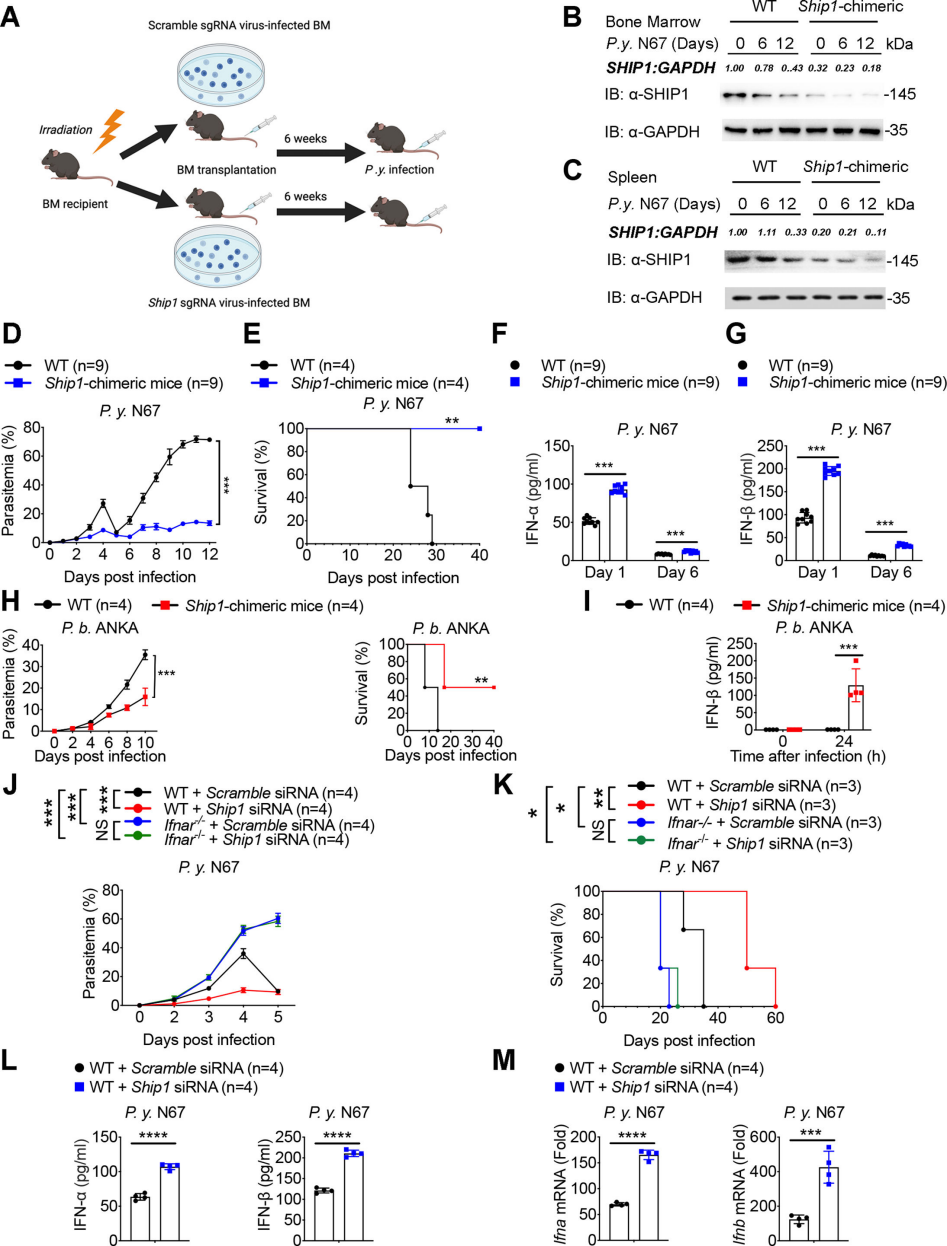
Disruption of *Ship1* gene increases IFN-I response and suppresses parasitemia. (**A**) Diagram of generating *Ship1* chimeric C57BL/6 mice. Bone marrow (BM) cells of recipient mice were depleted by radiation. BM cells with the *Ship1* gene disrupted using sgRNA technique were transplanted into the mice with depleted BM cells to generate *Ship1* chimeric mice. (**B and C**) Immunoblot analysis of SHIP1 expression in the BM (**B**) and the spleen (**C**) of WT and *Ship1*-chimeric mice on days 0, 6, and 12 post-infection with *P.y*. N67 infection. (**D**) Dynamics of parasitemia after infection of WT and *Ship1*-chimeric mice (*n* = 9) with *P.y*. N67 iRBCs (*i.p.,* 2 × 10^5^). (**E**) Survival of WT and *Ship1*-chimeric mice (*n* = 4) with *P.y*. N67 iRBCs (*i.p.,* 2 × 10^5^). (**F and G**) Protein levels of IFN-α (**F**) and IFN-β (**G**) in the serum of WT and *Ship1*-chimeric mice (*n* = 9) on day 1 and 6 after *P.y*. N67 infection. (**H**) Dynamics of parasitemia after infection of WT and *Ship1*-chimeric mice (*n* = 4) with *P.b*. ANKA iRBCs (*i.p.,* 2 × 10^5^), and survival of WT and *Ship1*-chimeric mice (*n* = 4) with *P.b*. ANKA iRBCs (*i.p.,* 2 × 10^5^). (**I**) the protein levels of IFN-β in the serum of WT and *Ship1*-chimeric mice (*n* = 4) at 24 hr after *P.b*. ANKA infection. (**J and K**) After 24 h of IOCV *scramble* siRNA or *Ship1* siRNA, dynamics of parasitemia (**J**) and survival (**K**) of WT and *Ifnar^-/-^* mice (*n* = 4) after infection with *P.y*. N67 iRBCs (*i.p.,* 2 × 10^5^). (**L and M**) Protein levels of IFN-α /IFN-β (**L**) in the serum and mRNA levels of *Ifna/b* (**M**) in the spleen of *scramble* siRNA and *Ship1* siRNA treated WT mice (*n* = 4) on day 1 after *P.y*. N67 infection. Data in (**D** to **M**) are means ± SEM of at least three independent experiments,^*^P < 0.05, ^**^P < 0.01, ^***^P < 0.001; NS, not significant (two-tailed Student’s *t* test).

To study the potential role of SHIP1 in host antimalarial immune response, we genetically ablated the *Ship1* gene in WT bone marrow (BM) cells using the CRISPR/Cas9 system and generated *Ship1*-chimeric mice to investigate the function of SHIP1 during malaria infection (Fig. 1A). We injected intraperitoneally (*i.p*.) 2 × 10^5^ of N67 infected red blood cells (iRBCs) into WT and *Ship1*-chimeric mice and evaluated SHIP1 expression in the BM and the spleen. Significantly reduced SHIP1 expressions were observed in the BM and spleen of the infected *Ship1*-chimeric mice, respectively (Fig. 1B and C). Parasitemia in WT mice infected with N67 rose rapidly for several days then reduced to low levels at day 5, before increasing again (3). The infected *Ship1*-chimeric mice showed significantly reduced parasitemia after N67 infection (Fig. 1D), and survived significantly longer than N67-infected WT mice which typically died on days 25–30 (3) (Fig. 1E), indicating that SHIP1 is detrimental for host antimalarial immunity.

IFN-I has been reported to be critical for stimulating stronger immune responses against N67 infection (3, 5, 21, 22), thus, we detected whether SHIP1 deficiency affects the production of IFN-I and found that N67C-infected *Ship1*-chimeric mice produced significantly higher levels of IFN-α and IFN-β in the serum than the infected WT mice (Fig. 1F and G). Meanwhile, the mRNA extracted from BMs of mice on days 1 and 6 post-N67 infection showed higher *Ifn-a/b* expression in *Ship1*-chimeric mice than the WT mice (Fig. S1A and B). Considering the negative role of *Ship1* in the NF-κB pathway (23), we also measured the production of pro-inflammatory cytokines such as TNF-α and IL-6, but detected no significant difference between *Ship1*-chimeric mice and WT mice after N67 infection (Fig. S1C and D). To investigate the effect of *Ship1* in other murine malaria models, we also generated *Ship1*-chimeric mice and infected them with *Plasmodium berghei* (*P.b*.) ANKA. The infected *Ship1*-chimeric mice showed reduced parasitemia and prolonged survival after ANKA infection (Fig. 1H). Also, we detected higher levels of IFN-β in the serum (Fig. 1I) and higher *Ifn-a/b* expression in the spleen (Fig. S1E and F) of *Ship1*-chimeric mice than the WT mice. We also determined whether *Ship1* mediates antimalarial immunity in non-virulent malaria, and observed lower parasitemia in *Ship1*-chimeric mice after the non-lethal strain *P.y*. 17XNL infection (Fig. S1G). These results suggest that SHIP1 inhibits IFN-I signaling during lethal or non-lethal malaria infection and genetic deletion of *Ship1* leads to a stronger IFN-I response and antimalarial immunity during malaria infection.

To investigate whether the resistance of *Ship1*-chimeric mice to N67 infection is dependent on the increased production of IFN-I, we infected WT and *Ifnar^−/^*^−^ mice with N67 after the tail vein injection (IOCV) of *scramble* small interfering RNA (siRNA) and *Ship1* siRNA, respectively; then, we detected lower parasitemia and longer survived in WT mice which handled with *Ship1* siRNA (Fig. 1J and K), and higher production of IFN-α/β in the serum and higher *Ifn-a/b* expression in the spleen than the infected WT mice which handled with *scramble* siRNA (Fig. 1L and M). Moreover, higher parasitemia and shorter survival time were observed in *Ifnar^−/^*^−^ mice after N67 infection, and the inhibition of *Ship1* on host antimalarial immunity was abolished in *Ifnar^−/^*^−^ mice (Fig. 1J and K). These results suggested that SHIP1-mediated inhibition on host antimalarial immunity is IFN-I-IFNAR dependent.

### SHIP1 negatively regulates malaria-induced IFN-I signaling

**Fig 2:**
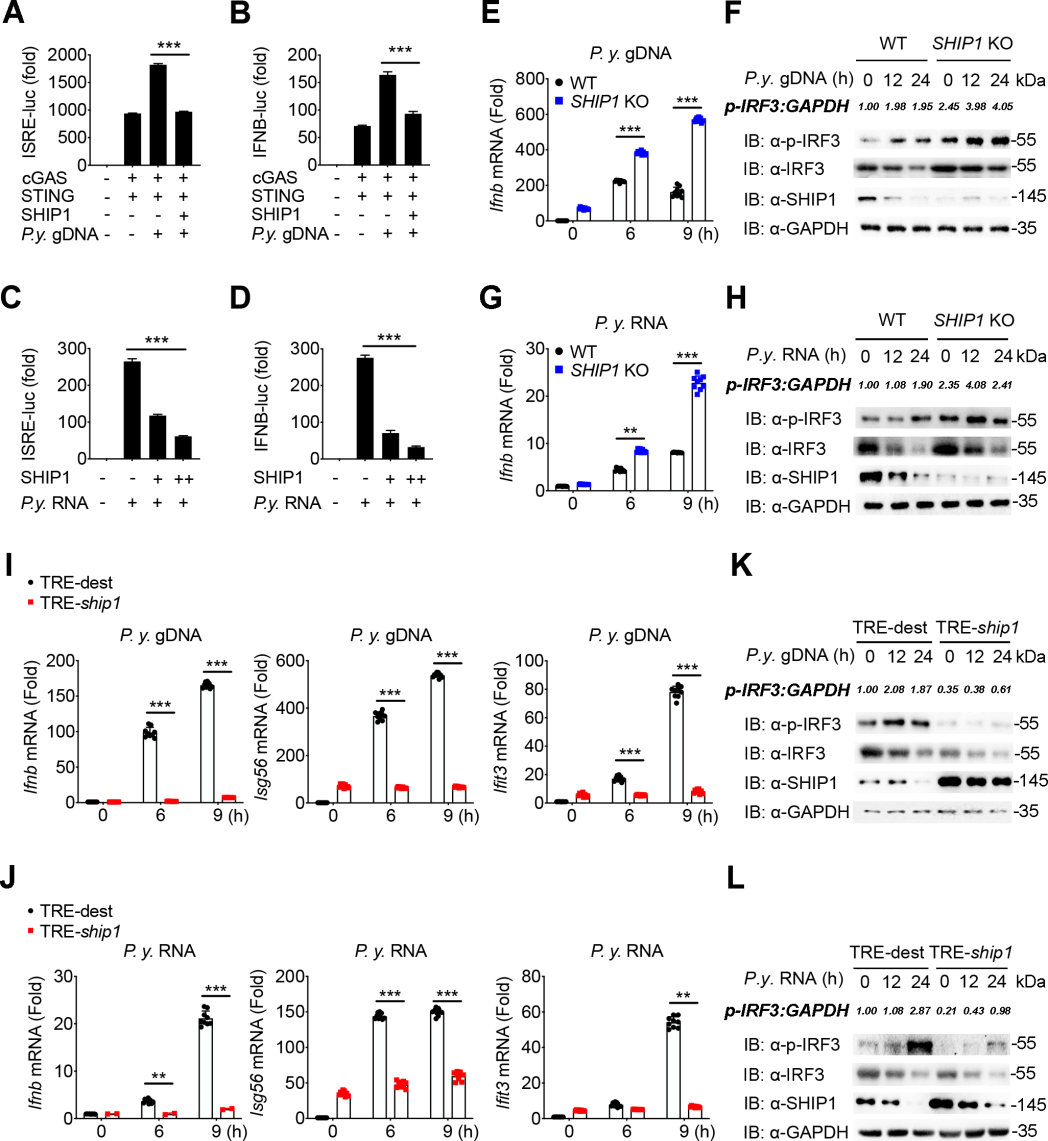
SHIP1 negatively regulates malaria parasite induced IFN-I signaling. (**A** to **D**) Luciferase activity in 293T cells transfected with ISRE (**A and C**) or IFN-β (**B and D**) promoter-driven luciferase reporters and together with plasmids encoding cGAS/STING (**A and B**) and increasing amount of plasmid expressing SHIP1. At 15 h after transfection, 293T cells were stimulated with *P.y*. gDNA (1 μg) (**A and B**) or RNA (1 μg) (**C and D**) for 9 h. (**E and G**) mRNA levels of *Ifnb* in WT and *SHIP1* KO RAW 264.7 cells at 0, 6, and 9 h after *P.y*. gDNA (1 μg) (**E**) or RNA (1 μg) (**G**) stimulation. (**F and H**) The protein levels of p-IRF3 in WT and *SHIP1* KO RAW 264.7 cells at 0, 12, and 24 h after *P.y*. gDNA (1 μg) (**F**) or RNA (1 μg) (**H**) stimulation. (**I and J**) mRNA levels of *Ifnb, Isg56,* and *Ifit3* in WT and *Ship1* doxy-inducible RAW 264.7 cells at 0, 6, and 9 h after *P.y*. gDNA (1 μg) (**I**) or RNA (1 μg) (**J**) stimulation. (**K and L**) The protein levels of p-IRF3 in *Ship1* doxy-inducible and WT RAW 264.7 cells at 0, 12, and 24 h after *P.y*. gDNA (1 μg) (**K**) or RNA (1 μg) (**L**) stimulation. Data in (**A-E**, **G**, **I**, and **J**) are means ± SEM of at least three independent experiments, ^**^P < 0.01, ^***^P < 0.001 (two-tailed Student’s *t* test).

Malaria infection results in host immune dysfunction, and a strong IFN-I response triggered by RNA polymerase III and melanoma differentiation-associated protein 5 (MDA5), binding of parasite DNA/RNA contributed to a decline of parasitemia in N67-infected mice (3, 24). However, the mechanism of IFN-I regulation during N67 infection remains unclear. To further investigate the role of SHIP1 in regulating malaria infection-induced IFN-I signaling, we purified genomic DNA (gDNA) (21, 25) and RNA from N67 parasites and performed luciferase reporter assay to measure IFN-I response in 293T cells after parasite gDNA or RNA stimulation. Ectopic expression of SHIP1 substantially inhibited ISRE-luc and IFN-β-luc activities triggered by *P.y*. gDNA or RNA (Fig. 2A to D).

We next genetically disrupted the *Ship1* gene in RAW 264.7 cells using CRISPR/Cas9 gene-editing technology and stimulated WT and SHIP1 knockout (KO) cells with *P.y.* gDNA or RNA for the indicated times. After 6 or 9 h stimulation with *P.y.* gDNA or RNA, significantly higher mRNA transcripts for *Ifnb* and ISGs, such as *Ifit3*, *Ifit2*, and *Isg56*, were detected in the SHIP1 KO cells than in WT cells (Fig. 2E and G; Fig. S2A and B). In addition, phosphorylated IRF3, which indicates activation of the IFN-I pathway, was more abundant in the SHIP1 KO cells after *P.y.* gDNA or RNA stimulation (Fig. 2F and H). Consistently, ELISA analysis revealed that SHIP1 deficiency promoted the production of IFN-β cytokine in the supernatants after *P.y.* gDNA or RNA stimulation(Fig. S2C and D). Taken together, these results demonstrate that SHIP1 deficiency significantly increases the expression of IFN-β and ISGs following malaria parasite gDNA/RNA stimulations *in vitro*, supporting that SHIP1 is a negative regulator of IFN-I signaling.

We next generated a Flag-tagged SHIP1 doxycycline (doxy)-inducible RAW 264.7 cells to examine the effects of SHIP1 overexpression on IFN-I response and found that doxy-mediated expression of SHIP1 resulted in lower mRNA levels of *Ifnb*, *Ifit3*, *Ifit2*, and *Isg56* after *P.y.* gDNA or RNA stimulation (Fig. 2I and J; Fig. S2E and F). Consistent with the results of lower mRNA levels, doxy-induced SHIP1 potently blocked the phosphorylation of endogenous IRF3 after *P.y.* gDNA or RNA stimulation (Fig. 2K and L). In addition, IFN-β protein was also decreased in the supernatants of SHIP1 doxy-induced cell culture compared with those of WT cells after *P.y.* gDNA or RNA stimulations(Fig. S2G and H). Collectively, these results support that SHIP1 negatively regulates malaria-induced IFN-I signaling.

We previously showed that MyD88-mediated IFN-I signaling in plasmacytoid dendritic cells (pDCs) played an important role in *P.y.* YM model (21). To verify whether *Ship1* inhibited the MyD88-mediated IFN in pDCs after N67 infection, we cultured pDCs by using FMS-like tyrosine kinase 3 ligand (FLT3L) to stimulate murine BM cells, and stimulated pDCs with parasite N67 gDNA for the indicated times after the handle of siRNA to knockdown the expression of SHIP1 (Fig. S2I). We observed the production of IFN-β in the supernatants (Fig. S2J) and the expression of *Ifn-a/b* (Fig. S2K and L), and found that there is no difference between *scramble* siRNA and *Ship1* siRNA treated cells. These results suggested that SHIP1 did not affect the MyD88-mediated IFN in pDCs after N67 gDNA stimulation.

### SHIP1 interacts with the IRF association domain of IRF3

**Fig 3:**
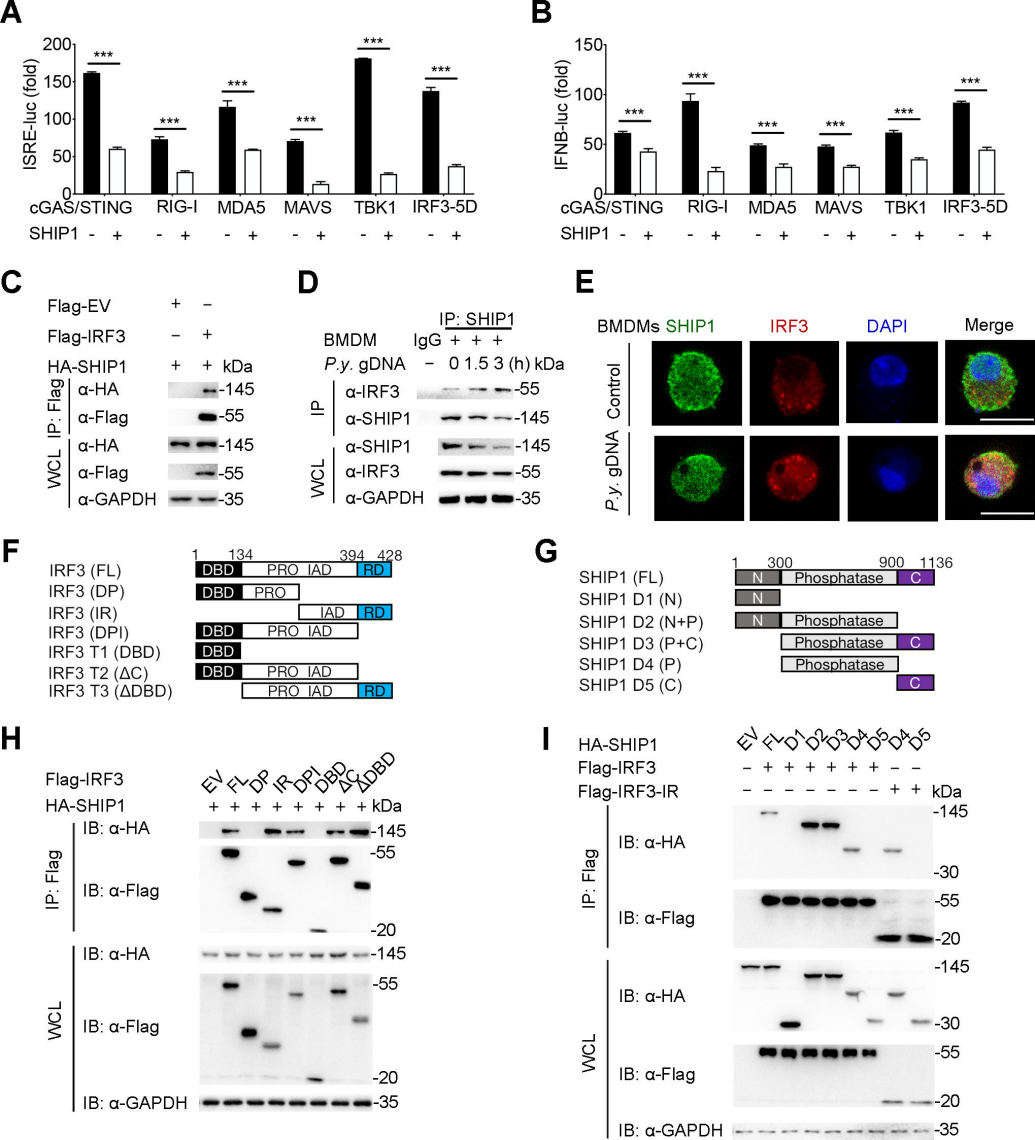
The phosphatase domain of SHIP1 interacts with IAD domain of IRF3. (**A and B**) 293T cells were transfected with the ISRE (**A**) or IFN-β (**B**) promoter-driven luciferase reporter and the indicated plasmids along with empty vector (black bars) or vector expressing HA-SHIP1 (white bars). (**C**) 293T cells were transfected with plasmids encoding HA-SHIP1 and Flag-IRF3, followed by IP using anti-Flag beads and immunoblot analysis with anti-HA antibody. (**D**) Extracts of BMDMs stimulated with *P.y.* gDNA (1 μg) for various times were subjected to IP with anti-IRF3 antibody and immunoblot analysis with indicated antibodies. (**E**) Distribution and cellular localization of IRF3 (red) and SHIP1 (green) were visualized in BMDMs with or without *P.y.* gDNA (1 μg) infection. The nuclear (blue) localization was shown at the same time. Scale bar = 10 μm. (**F and G**) Constructs containing different domains of IRF3 (**F**) and SHIP1 (**G**). (**H**) Co-IP and immunoblot analysis of 293T cells transfected with Flag-IRF3 and its indicated mutants along with vector encoding HA-SHIP1. (**I**) Co-IP and immunoblot analysis of 293T cells transfected with HA-SHIP1 and its indicated mutants along with vector encoding Flag-IRF3 or Flag-IRF3-IR fragment. Data in (**A**) and (**B**) are means ± SEM of at least three independent experiments, ^***^ P < 0.001 (two-tailed Student’s *t* test).

To investigate the molecular target of SHIP1 in IFN-I signaling pathways, we performed a luciferase reporter assay (8) and found that SHIP1 decreased the luciferase reporter activity induced by cyclic GMP-AMP synthase (cGAS)/stimulator of interferon genes (STING), RIG-I, MDA5, mitochondrial antiviral-signaling protein (MAVS), TANK-binding kinase 1 (TBK1), or IRF3-5D (a constitutively active mutant of IRF3) (Fig. 3A and B). Since SHIP1 can inhibit the IFN-I response by reducing IRF3 phosphorylation, we investigated whether SHIP1 could directly interact with IRF3 and other signaling molecules in the IFN-I pathway. Co-immunoprecipitation (co-IP) and immunoblot analysis showed that HA-tagged SHIP1 interacted with IRF3 and IRF3-5D (Fig. 3C; Fig. S3A), but not with cGAS, STING, RIG-I, MDA5, MAVS, or TBK1(Fig. S3A). To investigate the interaction between SHIP1 and IRF3 under physiological conditions, we stimulated RAW 264.7 cells, bone marrow-derived macrophages (BMDMs), and THP-1 cells with *P.y.* gDNA and collected the cell lysates at indicated times. We found that the endogenous association between SHIP1 and IRF3 increased upon *P.y.* gDNA stimulation (Fig. 3D; Fig. S3B and C). Furthermore, we investigated the co-localization of SHIP1 and IRF3 using confocal microscopy and showed slight co-localization between SHIP1 and IRF3 in BMDMs, and stronger co-localization after stimulation of parasite gDNA (Fig. 3E). These data indicate direct interaction between SHIP1 and IRF3.

IRF3 comprises the DNA binding domain (DBD), proline-rich region (PRO), IRF association domain (IAD), and reaction domain (RD) (17). To map the essential domains of IRF3 that interact with SHIP1, we generated several deletion constructs of IRF3 (Fig. 3F) and found FL (full length), IR (IAD+RD), DPI (DBD+PRO+IAD), ΔC (DBD+PRO+IAD), and ΔDBD (PRO+IAD+RD) of IRF3 interacted with the full-length SHIP1 protein, while IRF3 DP (DBD+PRO) and DBD did not associated with SHIP1 (Fig. 3H), indicating that the IAD domain of IRF3 is important for the IRF3-SHIP1 interaction. To further determine which domain of SHIP1 is responsible for its interaction with IRF3, we also generated several deletion constructs of SHIP1 (Fig. 3G). Co-IP and immunoblot analyses revealed that only the phosphatase domain of SHIP1 interacted with IRF3 (Fig. 3I). Consistently, the phosphatase domain of SHIP1 could interact with the IR (IAD+RD) domain of IRF3 (Fig. 3I). We also observed that the phosphatase domain of SHIP1 maintained the SHIP1-mediated inhibition of ISRE activity (Fig. S3D), indicating that the phosphatase domain of SHIP1 is critical for the suppression of IFN-I signaling. Taken together, these results suggest that SHIP1 inhibits IFN-I signaling by interacting with IRF3 through the phosphatase domain and IAD domains, respectively.

### SHIP1 promotes autophagic degradation of IRF3

**Fig 4:**
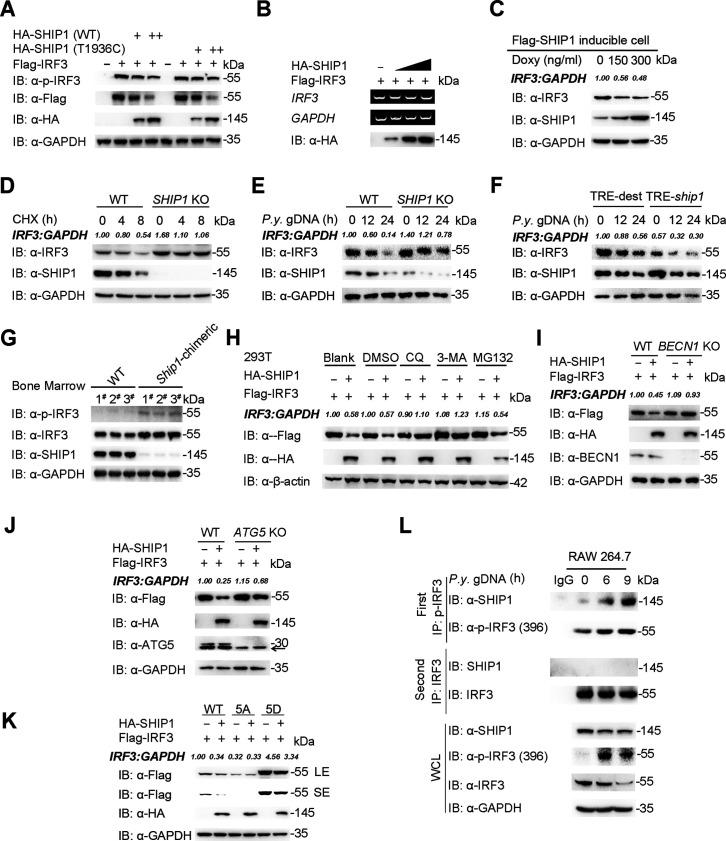
SHIP1 promotes autophagic degradation of IRF3. (**A**) Immunoblot analysis of extracts from 293T cells transfected with expression vector for Flag-IRF3 and increasing amount of vector for HA-SHIP1 or HA-SHIP1-T1936C mutant. (**B**) 293T cells were transfected with plasmids encoding Flag-IRF3 and increasing amount of HA-SHIP1. The mRNA level of *Irf3* was detected on agarose gel electrophoresis. (**C**) Flag-SHIP1 inducible RAW 264.7 cells were treated with indicated concentration of doxy for 12 h, and proteins were harvested for immunoblot analysis. (**D**) WT and *SHIP1* KO RAW 264.7 cells were treated with cycloheximide (CHX, 100 μg/mL) for indicated times, and the cell lysates were analyzed on immunoblot. (**E**) WT and *SHIP1* KO RAW 264.7 cells were stimulated with *P.y.* gDNA (1 μg) for various times, and the cell lysates were analyzed on immunoblot. (**F**) Flag-SHIP1 inducible RAW 264.7 cells were treated with indicated concentration of doxy for 12 h and stimulated with *P.y.* gDNA (1 μg) for various times. The lysates were analyzed with indicated antibodies. (**G**) Immunoblot analysis of the protein level of IRF3 and p-IRF3 in bone marrows of WT and *Ship1-*chimeric mice. (**H**) 293T cells were transfected with plasmids encoding Flag-IRF3 together with HA-SHIP1 plasmids, then treated with DMSO, chloroquine (CQ, 50 μM), 3-methyladenine (3-MA) (10 mM), or MG132 (10 μM) for 9 h. The cell lysates were analyzed on immunoblot. (**I and J**) WT, *ATG5* KO (**I**) and *BECN1* KO (**J**) 293T cells were transfected with plasmids encoding Flag-IRF3 and HA-SHIP1. Cell lysates were detected on immunoblot (arrow denotes nonspecific bands). (**K**) 293T cells were transfected with HA-SHIP1 and Flag-IRF3 or its mutants; cell lysates were analyzed on immunoblot; LE, long exposure; SE, short exposure. (**L**) Co-IP and immunoblot analysis of RAW 264.7 cells stimulated with *P.y.* gDNA for various times. Protein extracts were immunoprecipitated using anti-p-IRF3 and anti-IRF3 antibodies or IgG as a negative control and were analyzed on immunoblot using anti-p-IRF3, anti-IRF3 and anti-SHIP1 antibodies.

SHIP1 is known as a phosphatase that could decrease the protein phosphorylation level (12), and our results indicated that the phosphatase domain of SHIP1 is essential for its association with IRF3; thus, we hypothesized that the phosphatase activity of SHIP1 is required for its inhibition on IFN-I pathway. To test this possibility, we generated a construct encoding catalytically inactive SHIP1 with T1936C substitution, a mutant reported in 30% acute myeloid leukemia (AML) patients (26). 293T cells were transfected with plasmids encoding Flag-IRF3 together with increasing doses of WT SHIP1 or the inactive SHIP1 form (T1936C). Interestingly, the levels of IRF3 and p-IRF3 were decreased and the IRF3 protein levels were negatively correlated with that of SHIP1 protein no matter the cells were co-transfected with WT SHIP1 or the T1936C mutant (Fig. 4A). Moreover, the mRNA level of *Irf3* remained unchanged with increasing SHIP1, suggesting that SHIP1 promotes IRF3 protein degradation (Fig. 4B). Consistently, the phosphatase domain of SHIP1 alone could cause the degradation of IRF3 (Fig. S4A), suggesting SHIP1 mediated the activity of IRF3 in a catalytic activity-independent manner, but through its phosphatase domain. To exclude other proteins in the IFN-I pathway, we performed similar experiments and found that SHIP1 specifically degraded IRF3, but not RIG-I, MDA5, cGAS, STING, MAVS, or TBK1 (Fig. S4B to F).

Next, we ectopically expressed an increasing amount of Flag-SHIP1 with doxy induction and found that endogenous IRF3 was downregulated upon increasing expression of SHIP1 (Fig. 4C). Consistently, we observed that IRF3 turnover rates were reduced in SHIP1 KO cells in the presence of translational inhibitor cycloheximide (CHX) (27) (Fig. 4D). To assess whether SHIP1-mediated IRF3 degradation could be potentiated by malaria parasite DNA, we compared the degradation rates of IRF3 between WT and SHIP1 KO cells upon *P.y.* gDNA stimulation and found that KO of SHIP1 in RAW 264.7 cells decelerated endogenous IRF3 degradation elicited by *P.y.* gDNA stimulation (Fig. 4E). Conversely, doxy-induced expression of SHIP1 further accelerated the downregulation of endogenous IRF3 after *P.y.* gDNA stimulation (Fig. 4F). Furthermore, we observed much more protein amounts and phosphorylation of IRF3 in the BM of *Ship1*-chimeric mice than in WT mice (Fig. 4G). These results indicate that SHIP1 mediates IRF3 degradation upon *Plasmodium* DNA stimulation or infection.

The proteasome, lysosome, and autolysosome pathways are three major systems that eukaryotic cells used to control protein degradation (17). We next investigated which degradation system mediated the degradation of IRF3 by SHIP1 and found the degradation of IRF3 could be blocked by the autolysosome inhibitor chloroquine (CQ) (28) and autophagy inhibitor 3-methyladenine (3-MA) (17) but not by the proteasome inhibitor MG132 (Fig. 4H), suggesting that SHIP1 promoted autophagic degradation of IRF3. As previously reported (17), we also observed that IRF3 turnover rates were reduced in autophagy-related gene 5 (*ATG5*) and Beclin-1 (*BECN1*) KO cells (17) in the presence of CHX, in which the autophagy is significantly impaired (Fig. S4G and H). In addition, the degradation of IRF3 triggered by SHIP1 was almost abolished in *ATG5* and *BECN1* KO cells (Fig. 4I and J; Fig. S4I and J). Collectively, these results suggest that SHIP1 mediates IRF3 degradation through autophagy.

Given that IRF3 forms dimers after phosphorylation and translocates to the nucleus to activate downstream signaling (29), we next investigated which form (active or inactive) of IRF3 is regulated by SHIP1. We co-transfected 293T cells with Flag-IRF3-5D (a constitutively active mutant of IRF3) (30) or Flag-IRF3-5A (the substitution of these five S/T residues with A greatly impairs the function of IRF3) and HA-SHIP1 and observed that SHIP1 could promote the degradation of WT IRF3 and IRF3-5D, but not IRF3-5A (Fig. 4K), suggesting that SHIP1 mediated degradation of active IRF3 only. In addition, we performed a two-step IP assay and pulled down the phosphorylated and nonphosphorylated IRF3 separately after *P.y.* gDNA stimulation. The IP assay revealed the endogenous association between p-IRF3 and SHIP1 increased after *P.y.* gDNA stimulation, and there is no interaction between non-phosphorylated IRF3 and SHIP1 (Fig. 4L). Taken together, these data indicate that SHIP1 could interact with activated IRF3 and induce its autophagic degradation.

### SHIP1 interacts with cargo receptor NDP52 and facilitates the association between NDP52 and IRF3

**Fig 5:**
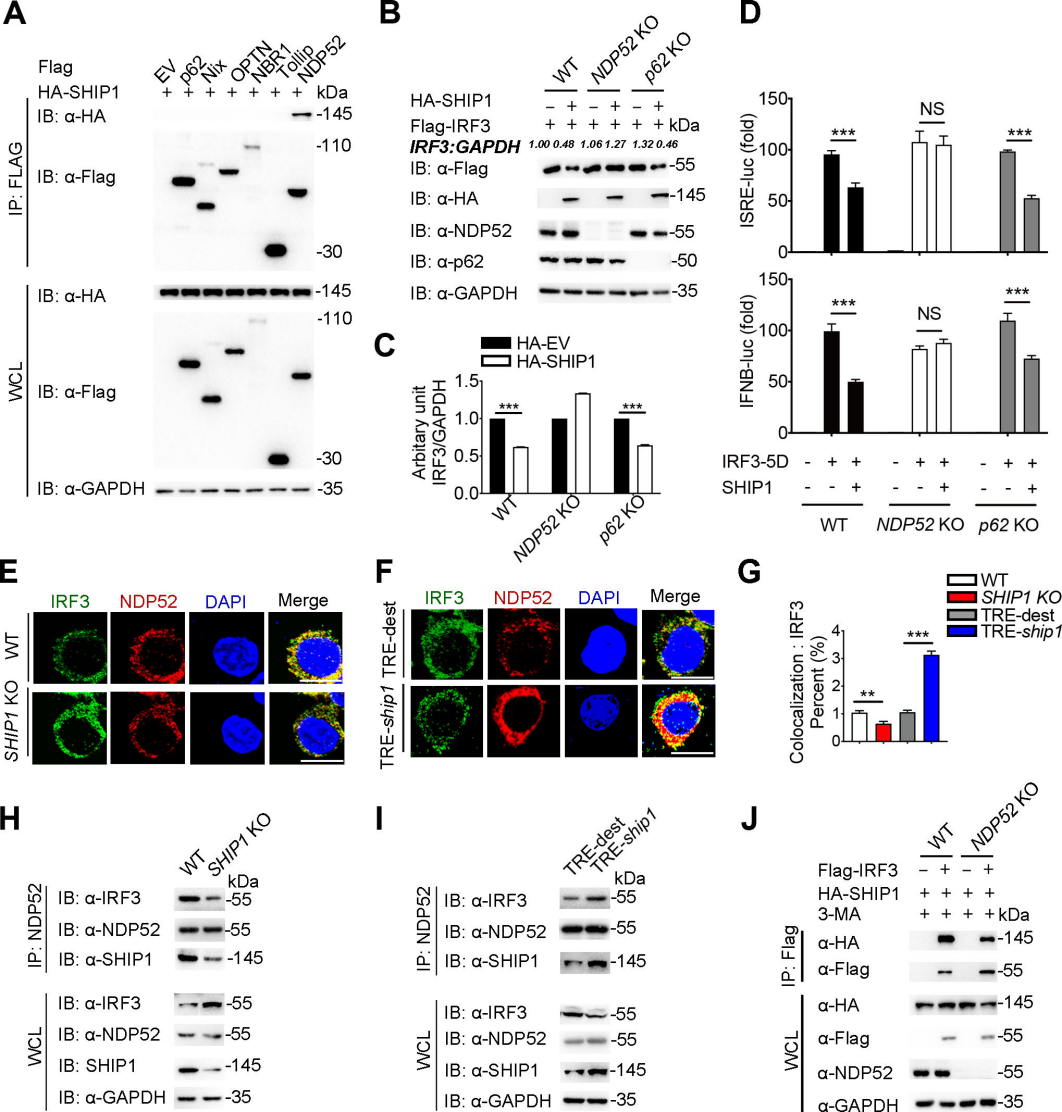
SHIP1 interacts with cargo receptor NDP52 and facilitates the association between NDP52 and IRF3. (**A**) 293T cells were transfected with vectors encoding HA-SHIP1 and the indicated Flag-tagged cargo receptors, followed by IP using anti-Flag beads and immunoblot analysis with anti-HA antibody. (**B**) WT, *NDP52* KO and *p62* KO 293T cells were transfected with plasmids encoding Flag-IRF3 and HA-SHIP1, the protein expression levels of IRF3 were detected on immunoblot. (**C**) Plots of scanned signals from (**B**). (**D**) WT, *NDP52* KO and *p62* KO 293T cells transfected with ISRE (the upper panel) or IFN- (the lower panel) promoter-driven luciferase reporter with or without the plasmids encoding *Ship1*; luciferase activity was detected at 24 h after IRF3-5D transfection. (**E and F**) Distribution and cellular localization of IRF3 (green) and NDP52 (red) were visualized in WT and *SHIP1* KO RAW 264.7 cells (**E**), or Flag-SHIP1 doxy-inducible RAW 264.7 cells (**F**). The nuclear (blue) localization was shown at the same time. Scale bar = 10 μm. (**G**) Quantitative analysis of signals from similar samples in (**E**) and (**F**). (**H**) Co-IP and immunoblot analysis of WT and *SHIP1* KO RAW 264.7 cells. Protein extracts were immunoprecipitated using anti-NDP52 antibody and analyzed on immunoblot using indicated antibodies. (**I**) Co-IP and immunoblot analysis of WT and Flag-SHIP1 doxy-inducible RAW 264.7 cells. Protein extracts were immunoprecipitated using anti-NDP52 antibody and analyzed on immunoblot using indicated antibodies. (**J**) Co-immunoprecipitation and immunoblot analysis of WT and *NDP52* KO 293T cells. Cells were transfected with plasmids encoding Flag-IRF3 and HA-SHIP1. Data in (**C*,* D and G**) are means ± SEM of at least three independent experiments, ^**^P < 0.01, ^***^P < 0.001; NS, not significant (two-tailed Student’s *t* test).

Accumulating evidence indicates that cargo receptors play essential roles in delivering substrates to the autophagosome for selective degradation (31). Thus, we next attempted to identify the potential cargo receptor responsible for the autophagic degradation of IRF3. Since SHIP1 is not a cargo receptor, we hypothesized that SHIP1 might bridge IRF3 to the cargo receptors for autophagic degradation. The co-IP assay showed that SHIP1 interacted with NDP52 but not other cargo receptors, such as p62, Nix, OPTN, NBR1, and Tollip (Fig. 5A), through its phosphatase domain (Fig. S5A). Therefore, SHIP1 interacted with both IRF3 and NDP52 through the phosphatase domain.

We next investigated whether NDP52 is involved in SHIP1-mediated autophagic degradation of IRF3. Indeed, SHIP1 could mediate IRF3 degradation in *p62* KO cells, but not in *NDP52* KO cells (Fig. 5B and C). Additionally, the inhibition of IFN-I activation by SHIP1 was abolished in *NDP52* KO cells (Fig. 5D). Likewise, CHX-chase assay results showed that the degradation of IRF3 was inhibited in *NDP52* KO 293T cells, compared with WT 293T cells (Fig. S5B and C). Taken together, these data suggest that SHIP1 contributes to IRF3 degradation through NDP52-mediated autophagy.

To further validate the role of SHIP1 in the association between IRF3 and NDP52. We performed confocal microscopy analysis and showed reduced co-localization puncta of IRF3-NDP52 when SHIP1 was KO in RAW 264.7 cells (Fig. 5E and G), and their co-localization was elevated upon doxy-induced SHIP1 overexpression (Fig. 5F and G). Furthermore, we observed that *SHIP1* KO dramatically disrupted the interaction of endogenous IRF3 and NDP52 (Fig. 5H). Conversely, doxy-inducible of SHIP1 in RAW 264.7 cells promoted the association between IRF3 and NDP52 (Fig. 5I). Consistently, the interaction of SHIP1 and IRF3 was reduced in *NDP52* KO 293T cells than in WT 293T cells (Fig. 5J). Altogether, these results indicate that SHIP1 facilitates the interaction between IRF3 and NDP52 for selective autophagic degradation.

### SHIP1 increases the K63-linked ubiquitination of IRF3 at the K313 site

**Fig 6:**
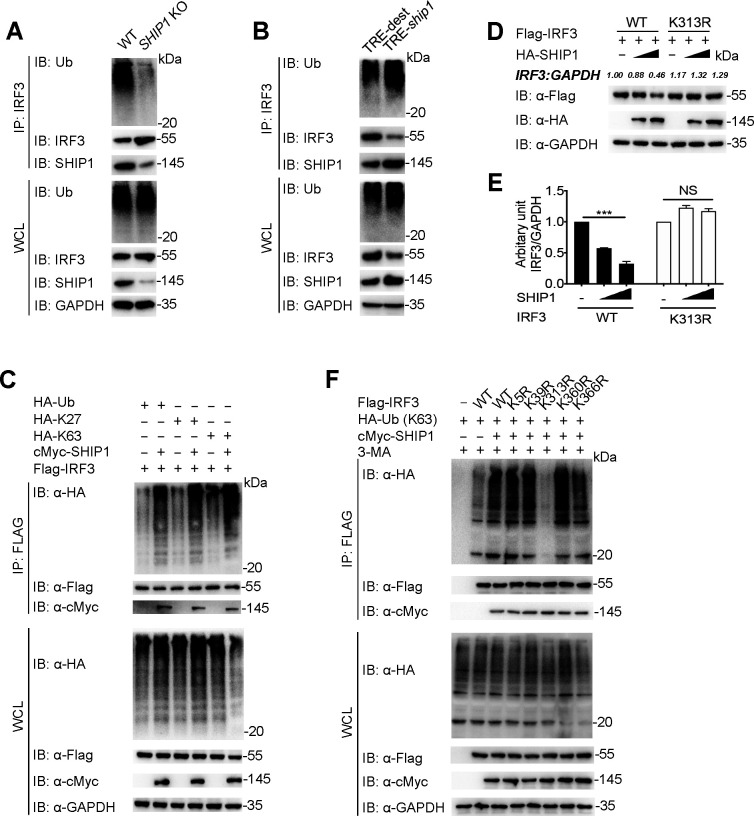
SHIP1 promotes K63-linked ubiquitination of IRF3 at K313 site. (**A**) Co-IP and immunoblot analysis of WT and *SHIP1* KO RAW 264.7 cells. Protein extracts were immunoprecipitated using anti-IRF3 antibody and analyzed on immunoblot using anti-IRF3 and anti-Ub antibodies. (**B**) Co-IP and immunoblot analysis of WT and Flag-SHIP1 doxy-inducible RAW 264.7 cells; protein extracts were immunoprecipitated using anti-IRF3 antibody and analyzed on immunoblot using anti-IRF3 and anti-Ub antibodies. (**C**) Lysates of 293T cells were transfected with plasmids expressing Flag-IRF3 and HA-tagged ubiquitin (Ub) and its indicated mutants, together with the empty vector or cMyc-SHIP1 expression vector. The cells were then treated with 3-MA (10 mM) and immunoprecipitated with anti-Flag beads and immunoblotted with anti-HA antibody. (**D**) 293T cells were transfected with plasmids encoding Flag-IRF3 or Flag-IRF3 (K313R) and HA-SHIP1; the lysates were analyzed on immunoblot 24 h after transfection. (**E**) Plots of scanned signals from (**D**). (**F**) 293T cells were transfected with plasmids expressing HA-K63-linked-Ub, Flag-IRF3 or its indicated mutants, together with the empty vector or cMyc-SHIP1 expression vector and treated with 3-MA (10 mM). The cell lysates were immunoprecipitated with anti-Flag beads and immunoblotted with anti-HA antibody. Data in (**E**) are expressed as means ± SEM of at least three independent experiments, ^***^P < 0.001; NS, not significant (two-tailed Student’s *t* test).

It is well known that cargo receptors recognize ubiquitin chains linked to substrates and target for autophagic degradation (17), and IRF3 has been reported to be degraded by inducing its connection with different kinds of ubiquitin chains, including K11, K27, K33, K48, and K63 ubiquitination after infections (17). We next investigated whether SHIP1 could affect the ubiquitination of IRF3 and found that *SHIP1* depletion remarkably decreased the poly-ubiquitination of endogenous IRF3 (Fig. 6A; Fig. S6A). Meanwhile, doxy-inducible of SHIP1 in RAW 264.7 cells increased the ubiquitination of IRF3 (Fig. 6B; Fig. S6B). To further investigate how SHIP1 regulates IRF3 ubiquitination, we transfected 293T cells with Flag-IRF3, cMyc-SHIP1, and HA-tagged K11-linked ubiquitin (K11-Ub), K27-Ub, K33-Ub, K48-Ub, or K63-Ub and found that SHIP1 specifically increased K27- and K63-linked ubiquitin chains on IRF3, but not others (Fig. 6C; Fig. S6C). Although mouse NDP52 and human NDP52 differ in the structures, with mouse NDP52 lacking the LIM-L domain (Fig. S6D) (32), both mouse and human NDP52 could bind to the ubiquitin molecules in 293T cells (Fig. S6E and F). We also found that the endogenous association between NDP52 and Ub in mouse BMDMs (Fig. S6G). Moreover, SHIP1 mediated stronger association between NDP52 and IRF3 was abolished when ubiquitination of IRF3 was blocked by ubiquitin inhibitor TAK-243 (MLN7243) (33) (Fig. S6H), indicating that SHIP1 promotes the ubiquitination of IRF3, thus facilitating the interaction between IRF3 and NDP52.

Several ubiquitination sites (K5, K39, K313, K360, and K366) of IRF3 have been reported previously (17). We next investigated the possible ubiquitination sites on IRF3 mediated by SHIP1. We transfected 293T cells with HA-SHIP1 and Flag-IRF3 or its mutants and observed that SHIP1-mediated degradation of IRF3 was abolished only when the IRF3 K313 site was mutated (Fig. 6D and E; Fig. S6I). Moreover, IRF3^K313R^ displayed a reduction in K63-linked ubiquitination but not K27-linked ubiquitination after co-transfected with HA-SHIP1 (Fig. 6F; Fig. S6J). In summary, these results reveal that SHIP1 induces the K63-linked ubiquitination of IRF3 at K313 and disrupts the stabilization of IRF3.

### SHIP1 is downregulated by IFN-I-induced miR-155-5p during malaria infection

**Fig 7:**
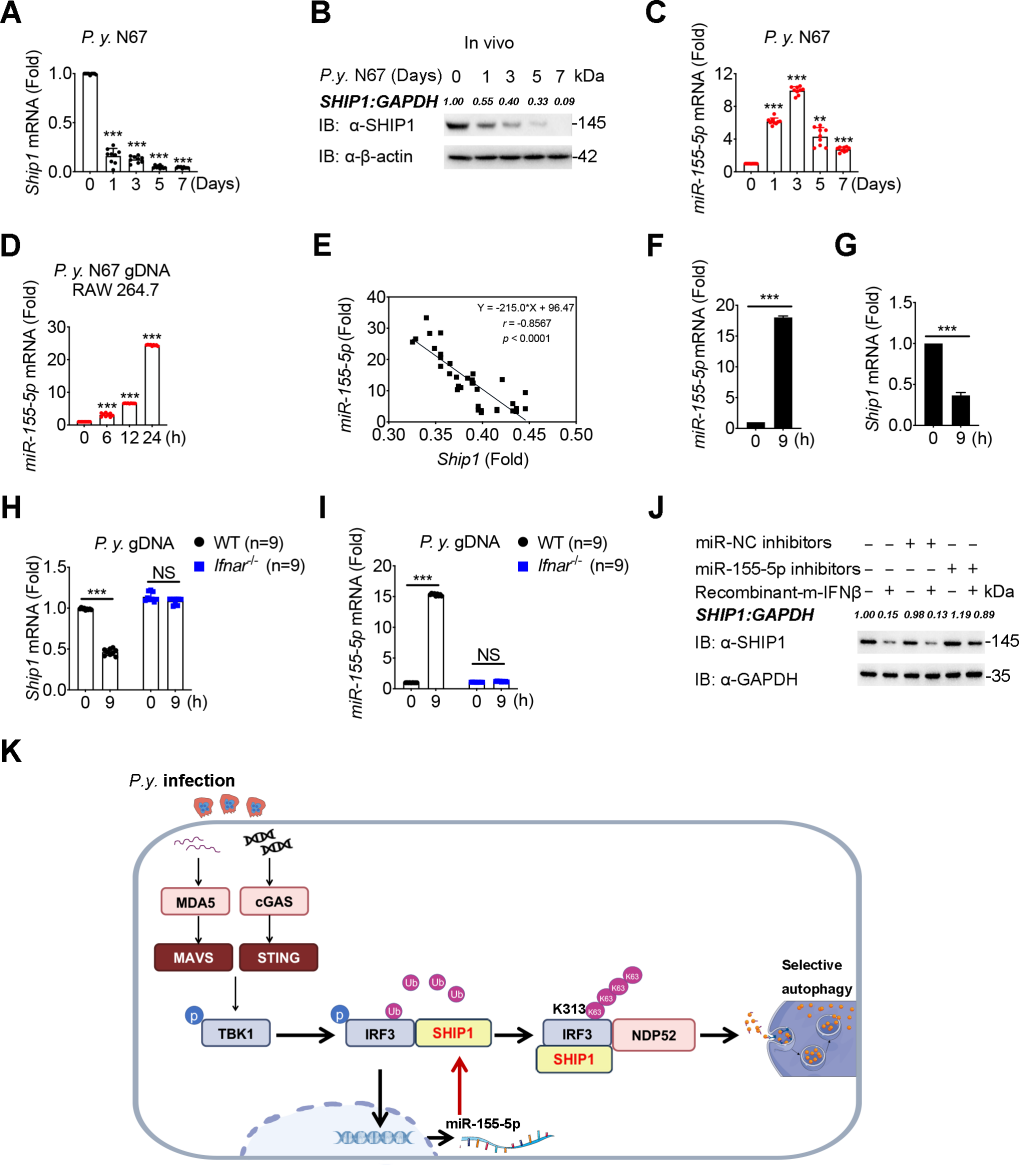
SHIP1 is downregulated by IFN-I-induced miR-155-5p during malaria parasite infection. (**A** to **C**) WT mice (*n* = 9) were injected *i.p*. with 2 × 10^5^ of *P.y.* N67 iRBCs. Spleens were collected at indicated times post infection. mRNA levels of *Ship1* in spleens were detected using qPCR (**A**); protein levels of SHIP1 in the spleens were analyzed on immunoblot (**B**); and mRNA levels of miR-155-5p in the spleens were detected using qPCR (**C**). (**D**) mRNA levels of miR-155-5p were detected in RAW 264.7 cells after stimulation with *P.y.* gDNA (1 μg) at the indicated times. (**E**) The correlation between *Ship* and miR-155-5p mRNAs in RAW 264.7 cells after stimulation with *P.y.* gDNA. (**F and G**) mRNA levels of miR-155-5p (**F**) and *Ship1* (**G**) were detected in RAW 264.7 cells after stimulation with mouse recombinant IFN-β (10 mM) at the indicated times. (**H and I**) mRNA levels of *Ship1* (**H**) and miR-155-5p (**I**) were detected in WT and *Ifnar^-/-^* BMDMs after stimulation of *P.y.* gDNA (1 μg) at the indicated times. (**J**) The protein levels of SHIP1 after IFN-β stimulation with or without miR-155-5p inhibitors in RAW 264.7 cells were analyzed on immunoblot. (**K**) A proposed working model of SHIP1 regulating IFN-I response. During the malaria parasite infection, SHIP1 binds to phosphorylated IRF3 and promotes K63-linked poly-ubiquitination of IRF3 at K313, which stabilizes the interaction of IRF3 and its selective cargo receptor NDP52. The interactions of SHIP1, IRF3, and NDP52 enhance activated IRF3 autophagic degradation and turns down IFN-I responses against malaria parasite infection. Meanwhile, IRF3-dependent IFN-I activated by parasite gDNA and RNA induce miR-155-5p to downregulate SHIP1, which serves as a negative feedback loop between IFN-I signaling and autophagy. Data in (**A, C, D, F, G, H and I**) are means ± SEM of at least three independent experiments, ^***^P < 0.001; NS, not significant (two-tailed Student’s *t* test).

Finally, we investigated whether SHIP1 expression could also be regulated in response to malaria infection. Real-time PCR and western blot analyses revealed strong downregulation of SHIP1 at both the mRNA and protein levels after *P.y.* N67 infection (Fig. 7A and B). To further confirm the expression levels of SHIP1, we also stimulated RAW 264.7 cells or BMDMs with *P.y.* gDNA or RNA and observed similar trends of reduction of SHIP1 mRNA in RAW 264.7 cells or BMDMs after *P.y.* gDNA or RNA stimulation (Fig. S7A to D), which indicated that the expression of SHIP1 is restrained upon malaria infection. Since human malaria models may differ from the murine malaria models, we also used human *P.f.* 3D7 gDNA and RNA to stimulate THP-1 cells, and found a similar trend of downregulation in the mRNA levels of *Ship1* after *P.f.* 3D7 gDNA or RNA stimulation (Fig. S7E and F), as in the murine models.

microRNAs (miRNAs) are small endogenous RNAs that are critical regulators of gene expression and promising candidates for biomarker development (34). About 100 miRNAs are altered during malaria infection (35). We detected several miRNAs that are most correlated with malaria (36), including miR-25-3p, miR-146a-5p, and miR-155-5p, for the decreased trends of *Ship1*, and found that miR-155-5p has the most corresponding trends with *Ship1* (Fig. S7G to M). Since SHIP1 was reported to be a direct and functionally significant target of miR-155-5p in the context of viral and bacterial infections (37), we next sought to determine whether miR-155-5p is involved in the downregulation of SHIP1 during malaria infection. Indeed, the luciferase reporter assay showed that miR-155-5p mimics blocked the relative activity of the reporter luciferase of *Ship1*-3′UTR (Fig. S7N), confirming the direct interaction between miR-155-5p and *Ship1* gene. In addition, the expression of miR-155-5p mimics decreased the expression of SHIP1 protein in 293T cells, while miR-155-5p inhibitors enhanced the protein level of SHIP1 (Fig. S7O and P).

Next, we asked whether miR-155-5p can inhibit SHIP1 during malaria parasite infection. A significant increase in the miR-155-5p expression levels during *P.y.* N67 infection (Fig. 7C) and nucleic acid components stimulation (Fig. 7D; Fig. S7G to I) was detected, and there was an inverse correlation between the miR-155-5p and the *Ship1* mRNA levels during malaria infection (Fig. 7E), indicating that SHIP1 could be downregulated by miR-155-5p after *P.y.* N67 infection. Furthermore, we observed that the induction of miR-155-5p and the downregulation of SHIP1 (Fig. 7F and G) were abolished in *Ifnar^−/^*^−^ BMDM after *P.y.* gDNA stimulation, compared with WT BMDM (Fig. 7H and I), suggested that the change of miR-155-5p and SHIP1 was controlled by downstream of IFN-I. Moreover, the decrease in SHIP1 triggered by IFN-β stimulation was restored when the cells were co-transfected with miR-155-5p inhibitors (Fig. 7J). Altogether, these results suggest that SHIP1 is downregulated by IFN-I-induced miR-155-5p during malaria infection and serves as a negative feedback mechanism between IFN-I signaling and autophagy.

## DISCUSSION

IFN-I must be tightly controlled to maintain a balanced immune response between defense and homeostasis in infectious diseases (38, 39). Mounting evidence has shown that multiple regulators are involved in the regulation of IFN-I production during infection, and regulators’ expressions are also modulated by the activation of IFN-I signaling as well (40, 41). Many negative regulators, such as Tetherin, NLRC5, NLRP11, and RTP4, are induced after infection, to feedback and inhibit the production of IFNs (5, 32, 42, 43). The induction of negative regulator is important for organisms to maintain immune homeostasis, which also plays key roles in the escape mechanism of pathogens (44). However, other negative molecules are downregulated to relieve their inhibition on IFN-I signaling and enhance host immune responses against infection (45, 46). Here, we discover another IFN-I response regulator SHIP1 and show that SHIP1 is downregulated by IFN-I-mediated miR-155-5p. Downregulation of SHIP1 increases phosphorylation and stability of IRF3 and IFN-I production, leading to improved host antimalarial immunity after N67 parasite infection. Although reduction of SHIP1 after *P.y.* N67 infection could ameliorate degradation of IRF3 and enhance IFN-I in WT mice, which still cannot generate sufficient immune responses against malaria infection. Our data showed that only disruption of *Ship1* at an early time of infection could enhance IFN-I response timely and protect host against *P.y.* N67 parasites, which is consistent with our previous study that only the-early-stage-IFNs can protect the organism (25, 47). The timing of production of IFNs is critical for antimalarial immune responses (3), and that is why we observed more resistance in *Ship1*-chimeric mice in comparison to WT mice during malaria infection. During the N67 infection, the production of IFN-I peaks at day 1 after infection, which is consistent with previous publication (3, 22), and decreases to a basal line at the late stage of infection. Although the amounts of IFN-α/β are low on day 6, *Ship1*-chimeric mice still generate high levels of IFN-α/β than the WT mice, which further suggests the inhibition of SHIP1 on the production of IFN-I throughout the course of malaria infection.

Decades of studies have revealed that SHIP1 is a multifunction protein that carries out its tasks by distinct means (48). Early studies mainly focused on SHIP1’s function in bone biology, cancer, and mucosal inflammation (49). SHIP1 promotes the development of bone through its nonenzymatic functions (50); meanwhile, the inhibition of enzymatic activity of SHIP1 enhances the survival in cancer cells (26). The loss of SHIP1 function causes severe inflammation in human Crohn’s disease (51). Recently, accumulating evidence has shown that SHIP1 affects cellular behaviors in innate and adaptive immune cells by dephosphorylating the PI3K (48). A study in AML patients showed that different SHIP1 mutants influence its phosphatase activity, and the AML mice increased their lifespan after lentiviral-mediated overexpression of SHIP1 (26). In autoimmune diseases, such as systemic lupus erythematosus and rheumatoid arthritis, SHIP1 functions as a critical negative regulator of TLR3-mediated IFN-β production through affecting the localization and activity of TBK1 (52). However, different from the previous studies, here we demonstrated that SHIP1 mainly targets on IRF3, but not TBK1, after *P.y.* N67 infection. Although SHIP1 may dephosphorylate IRF3 by its phosphatase activity, it can also regulate the stability of IRF3 through its nonenzymatic activity and promotes selective autophagic degradation of IRF3 to inhibit IFN-I production during malaria infection. Although gDNA stimulation lowers the expression of *Ship1*, its association with IRF3 increased after stimulation, which indicated that more SHIP1 interacted with activated IRF3 in the cytoplasm, even though total SHIP1 may be downregulated by miR-155 or undergo degradation upon malaria infection. Besides IFN-I signaling, NF-κB pathway is also critical during malaria infection. Parasite protein circumsporozoite protein was reported to interfere with the NF-κB pathway by outcompeting NF-κB nuclear import (53). In addition, the activation of heat shock protein 70 (Hsp70) and NF-κB could be suppressed by Hemozoin during severe malarial anemia (54). Thus, *Artemisia annua* L.’s active ingredient, artemisinin, has been widely used for malaria therapy by its numerous bioactivity including inhibition of the NF-κB pathway (55). SHIP1 is also well known for its regulation on NF-κB signaling by targeting MyD88 (48). However, SHIP1 did not affect NF-κB pathway and production of pro-inflammatory cytokines during malaria parasite N67 infection in our study. Our data suggested that the main role of SHIP1 in antimalarial immunity was dependent on its regulation of IFN-I signaling, which indicates that multiple innate immune signaling pathways contribute synergistically to host antimalarial immunity.

Malaria is typically transmitted to humans by the bite of female *Anopheles* mosquito, which carriers the *Plasmodiu*m parasites (56). The transcription of *Ship1* has been reported to be downregulated in *P.f.* infected patients (15). Due to the limitations in obtaining human samples, we used the murine malaria model for this study. We observed *Ship1*-chimeric mice can obtain more protection in both the nonlethal murine malaria stain (*P.y.* N67 and 17XNL) and the lethal strain (*P.b.* ANKA) models. Significantly higher levels of IFN-I responses, lower parasitemia, and better survival rates were observed in *Ship1*-chimeric mice than in WT mice. Altogether it verified SHIP1-mediated IFN-I immunity is not only specific for *P.y. nigeriensis* but also applicable to the other virulent and nonvirulent malaria.

Phosphorylation of IRF3 is a critical step for IFN-I signaling activation. IRF3 locates in the cytoplasm in the unstimulated state and is phosphorylated at specific serine residues which allow its dimerization and nuclear translocation to initiate IFN-I signaling upon infection. IFN-I needs to be tightly controlled to maintain host immune balance; thus, the post-translational modification of IRF3 plays a critical role in determining the immune state against infection. We previously reported that the phosphorylated IRF3 could undergo K27-linked ubiquitination, which led to the NDP52-mediated autophagic degradation and the immune suppression of IFN-I signaling after Sendai virus infection (17). Different from the previous study, here we demonstrate that SHIP1 also modulates the stability of activated IRF3 through K63-linked ubiquitination of IRF3 at K313, which is also a recognition signal for NDP52-dependent selective autophagic degradation resulting in inhibition of antimalarial immunity. These results indicate that different types of ubiquitination control IRF3 activity and stability through autophagic degradation and lead to distinct outcomes during different microbe infections. However, since SHIP1 is not an E3 ligase, it may regulate certain E3 ligase of IRF3 and then participate in the regulation of IRF3’s ubiquitination. Whether this K63-linked ubiquitination of IRF3 mediated by SHIP1 also occurs in other infections requires further investigations.

Autophagy is a conserved degradation pathway that disassembles and recycles dysfunctional cellular components (57). CQ, as a traditional antimalarial drug, could directly target host’s autophagy system in malaria, which also has additional functions, including antivirus, antibacteria, antiprotozoan, antiautoimmunity, and anticancer effects (58). However, the mechanism of CQ in malaria and other diseases is not understood well (58). Accumulating evidence has revealed the crosstalk between autophagy and IFN-I signaling in infectious diseases. During viral infection, coiled-coil domain-containing protein 50 (CCDC50) functions as autophagy receptor and negatively regulates the IFN-I by promoting the autophagic degradation of RIG-I/MDA5 (59). An Epstein-Barr virus encoding BCL2 homolog (BHRF1) inhibits IFN-β induction by targeting the mitochondria and blocking the nuclear translocation of IRF3 (60). Recent study showed that MGF-505-7R promotes the expression of the autophagy related protein ULK1 to degrade STING in African swine fever virus infection model (61). However, autophagy is a double-edged sword in infectious disease, and the relationship between autophagy and IFN-I is still not clarified. Autophagy can induce the production of IFN-I or degrade some important signal molecules in a direct or indirect way, thus dissecting the interactions between autophagy and IFN-I signaling in infectious diseases may help us better understand the underlying mechanism (19). Here, we demonstrate that SHIP1 enhances the association between cargo protein NDP52 and IRF3 and promotes the autophagic degradation of IRF3, leading to inhibition of IFN-I production and suppression of immune responses against N67 infection. Our study suggests targeting autophagy could be an effective interference for modulating antimalarial immunity.

Noncoding RNAs, including miRNAs and long noncoding RNAs, have emerged as key regulators of gene expression in immune cell development and function. Their expression is altered in different physiological and disease conditions, hence making them attractive targets for the understanding of disease etiology and developing complementary control strategies (36). The previous study summarized the miRNAs in malaria patients and highlighted several miRNAs that are involved in different stages of malaria development, such as miR-146a-5p, miR-25-3p, miR-155-5p, and others (62). miR-155-5p was reported to contribute to the pathogenesis of cerebral malaria by regulating endothelial activation, microvascular leak, and blood-brain barrier, and then aggravate the malaria process (63). In our study, we revealed that SHIP1 was targeted and downregulated by miR-155-5p, which was consistent with a previous study (62). However, we still do not know whether the proteasome or the autolysosome system is used to control SHIP1 degradation. Since 3-MA was reported as an potential antagonist of SHIP1 (63), the degradation of SHIP1 during malaria parasite infection may be influenced by autophagy in some way.

In summary, we identify a novel role for SHIP1 in negative regulation of antimalarial immunity. Based on our findings, we proposed a working model to illustrate how SHIP1 suppresses IFN-I production during malaria infection. After *Plasmodium* infection, IRF3-dependent IFN-I is activated by parasite gDNA and RNA (3). However, SHIP1 binds to phosphorylated IRF3 to promote K63-linked poly-ubiquitination on IRF3 at K313, which stabilizes the interaction of IRF3 and its selective cargo receptor NDP52, and targets the activated IRF3 for autophagic degradation. Degradation of IRF3 thus turns off IFN-I responses against malaria infection. Meanwhile, SHIP1 is also regulated by IFN-I-induced miR-155-5p, which serves as a negative feedback loop controlling the crosstalk between IFN-I and autophagy (Fig. 7K). Inhibition of SHIP1 activity will stabilize pIRF3, enhance early IFN-I response, and suppress parasitemia. SHIP1 could be a potential therapeutic target for the treatment of malaria and other infectious diseases.

## MATERIALS AND METHODS

### Cell line and culture conditions

HEK293T, RAW 264.7, and THP-1 cells were cultured in DMEM or RPMI1640 (Corning, Invitrogen, Carlsbad, CA, USA) containing 10% fetal bovine serum (FBS) (Gibco, New York, NY, USA) incubated in a 5% CO_2_ chamber (Thermo Fisher Scientific, MA, USA). BMDMs were derived from the BM of 18–20 g C57BL/6 mice (Guangdong Medical Laboratory Animal Center, Guangzhou, Guangdong, China) and cultured for 6–8 days with DMEM containing 10% FBS and the supernatant from L929 cells (mouse fibroblast cells). Culture for pDCs: BM cells were isolated from the tibia and femur and cultured in RPMI1640 medium with 10% FBS, 1% penicillin-streptomycin, and 200 ng/mL Flt3L for pDCs.

### Generation of *SHIP1* stable expression and knockout cell lines

The retroviral vectors were co-transfected with an expression plasmid for the vesicular stomatitis virus G protein into the HEK293T cell line. The medium was changed the following day, and the viral containing supernatant was collected 48 h after transfection, filtered through a 0.45-μm filter and subsequently used to infect cells with polybrene (10 μg/mL). For SHIP1 ectopic expression, lentiviral particles were produced by transfecting HEK293T cells with FG-EH-DEST-SHIP1, VSVG, and Δ8.9. RAW 264.7 cells were infected by incubation with retrovirus-containing supernatant for 48 h. For *ATG5* KO, *BECN1* KO, *NDP52* KO, and *p62* KO cells, target sequences were cloned into pLentiCRISPRv2 by cutting with BsmBI. Transduced cells were purified by puromycin selection. *SHIP1* KO RAW 264.7 cells and BM cells were generated using the CRISPR/Cas9 system, and the sequences of target sgRNA are as follows: *Ship1*-sgRNA (CACCGACGCAGAGTGCGTAGGCCCG).

### Extraction of malarial gDNA and RNA

Parasite-infected mice blood was collected in saline solution and filtered to deplete white blood cells. Parasites were spun down after RBC lysis buffer treatment, and lysate was incubated with buffer A (150 mM NaCl, 25 mM EDTA, 10% SDS, and protein kinase) overnight. gDNAs were isolated using phenol/chloroform, and RNAs were isolated using TRIzol reagent (Invitrogen, CA, USA).

### siRNA treatment *in vivo*

RNAi-mediated silencing in mice *in vivo* Ready siRNAs was mixed with Invivofectamine 3.0 liposomes (Invitrogen, CA, USA) following the manufacturer’s instructions (TsingkeBiotechnologyCo. Ltd, Guangzhou, Guangdong, China) and injected intravenously in a volume of 100 µL at a dose of 5 mg/kg. Mice were infected with *P.y*. N67 (2 × 10^5^ iRBCs) at 48 h after siRNA treatment. *Ship1*-siRNA (GGCTCAGAATCTACCAACA).

### Animal and chimeric model

The packaged virus of sg-v2 or sg-m-*ship1* was produced by 8.9/VSVG lentivirus expression system and infected BM cells of 6- to 10-week-old donor female mice. Recipient mice aged 8–10 weeks (female, WT C57BL/6) were irradiated with 900 cGy and randomly divided into two groups. They received BM cells (5 × 10^6^) infected with sg-v2 or sg-m-*ship1*, respectively. The chimeric mice were housed in the SPF animal room and the BMs or spleens from two groups were collected to detect the level of SHIP1 after 6–8 weeks.

### Malaria parasite infection procedures

The parasites *P.y.* N67, 17XNL, *P.f.* 3D7, and *P.b.* ANKA were initially obtained from the Malaria Research and Reference Reagent Resource Center (MR4, https://www.beiresources.org/About/MR4.aspx). The parasites *P.y.* N67 and *P.b.* ANKA have been previously described (24). For N67 infection, 2 × 10^5^ iRBCs suspended in 200 μL PBS from the donor mice were injected *i.p.* into experimental mice (8-week-old female C57BL/6 mice were acquired from the Experimental Animal Centre of Southern Medical University). For ANKA infection, 2 × 10^5^ iRBCs suspended in 200 μL PBS from the donor mice were injected *i.p.* into experimental mice. For *P.y.* 17XNL infection, 1 × 10^5^ iRBCs suspended in 200 μL PBS from the donor mice were injected *i.p.* into experimental mice. All mouse-related procedures were performed according to experimental protocols authorized by the Southern Medical University Animal Care and Use Committee (SMUL2019243). Detailed information could be found in the Supporting Information.

### Statistical analysis

The data of all quantitative experiments are presented as mean±SEM of at least three independent experiments. Curve data were assessed by GraphPad Prism 8.0 (San Diego, California, USA). And comparisons between groups for statistically significant differences were analyzed with a two-tailed Student’s *t* test. The statistical significance was defined as *P* < 0.05.

## SUPPLEMENTAL MATERIAL

Fig S1 mbio.03512-22-s0001.tif SHIP1 is detrimental for antimalarial immunity.

Fig S2 mbio.03512-22-s0002.tif SHIP1 is a negative regulator of type I IFN signaling in host antimalarial immunity.

Fig S3 mbio.03512-22-s0003.tif SHIP1 interacts with IRF3, but not with other molecules in type I IFN pathways.

Fig S4 mbio.03512-22-s0004.tif SHIP1 mediates autophagic degradation of IRF3.

Fig S5 mbio.03512-22-s0005.tif SHIP1 promotes IRF3 interacts with NDP52.

Fig S6 mbio.03512-22-s0006.tif SHIP1 promotes the K63-linked ubiquitination of IRF3.

Fig S7 mbio.03512-22-s0007.tif Malaria infection induces the degradation of Ship1 by upregulating miR-155-5p.

Legends and additional experimental details mbio.03512-22-s0008.docx The legends of supplemental figures; supplemental materials and methods.

ASM does not own the copyrights to Supplemental Material that may be linked to, or accessed through, an article. The authors have granted ASM a non-exclusive, world-wide license to publish the Supplemental Material files. Please contact the corresponding author directly for reuse.
