# Supplementary figures and images for: SHIP1 modulates antimalarial immunity by bridging the crosstalk between type I IFN signaling and autophagy
Source: mBio. 2023 Jun 27;14(4):e03512-22. doi: 10.1128/mbio.03512-22 (PMC10470592; doi:10.1128/mbio.03512-22)

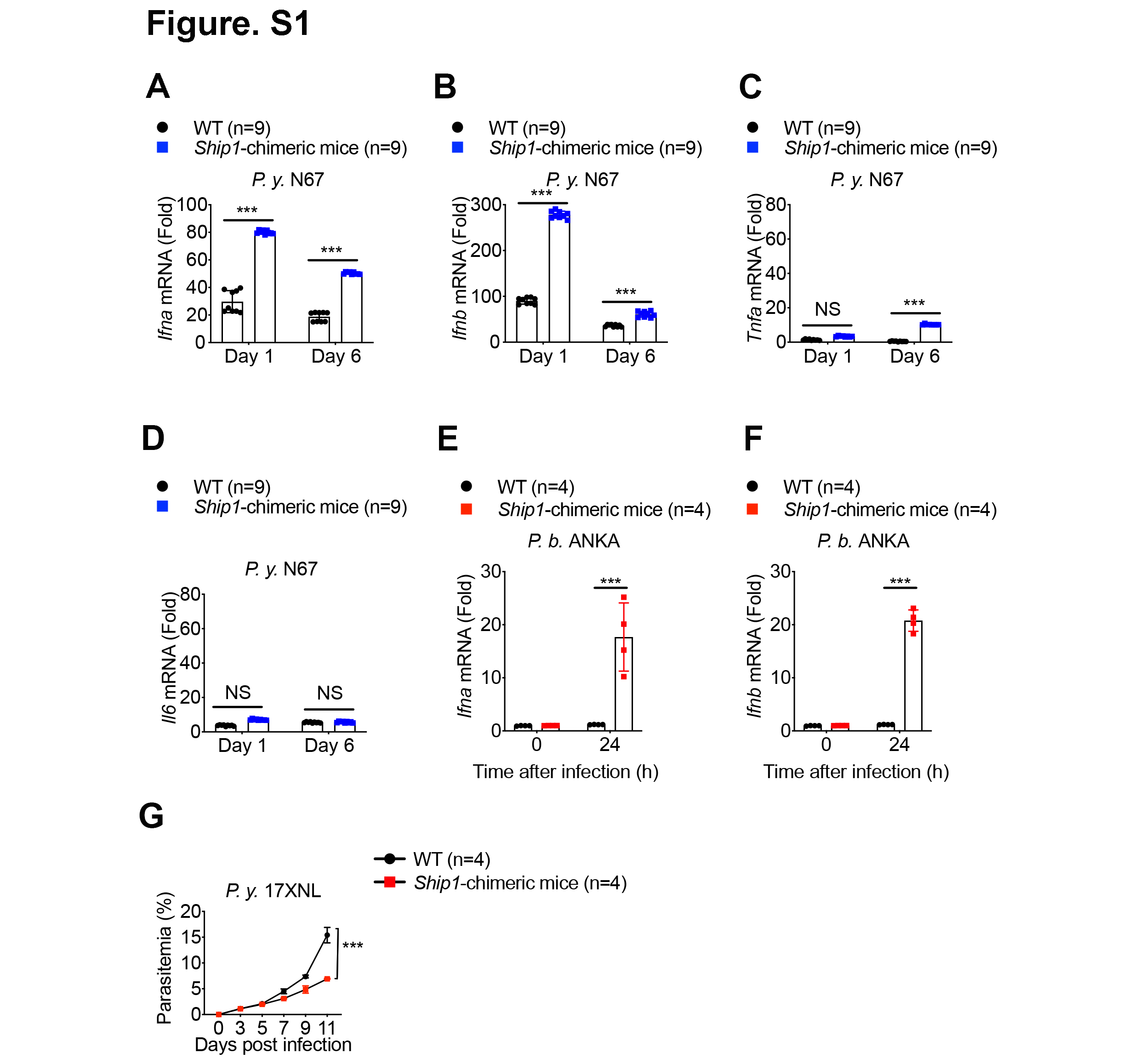

Supplement: Fig S1 — SHIP1 is detrimental for antimalarial immunity. [file mbio.03512-22-s0001.tif]

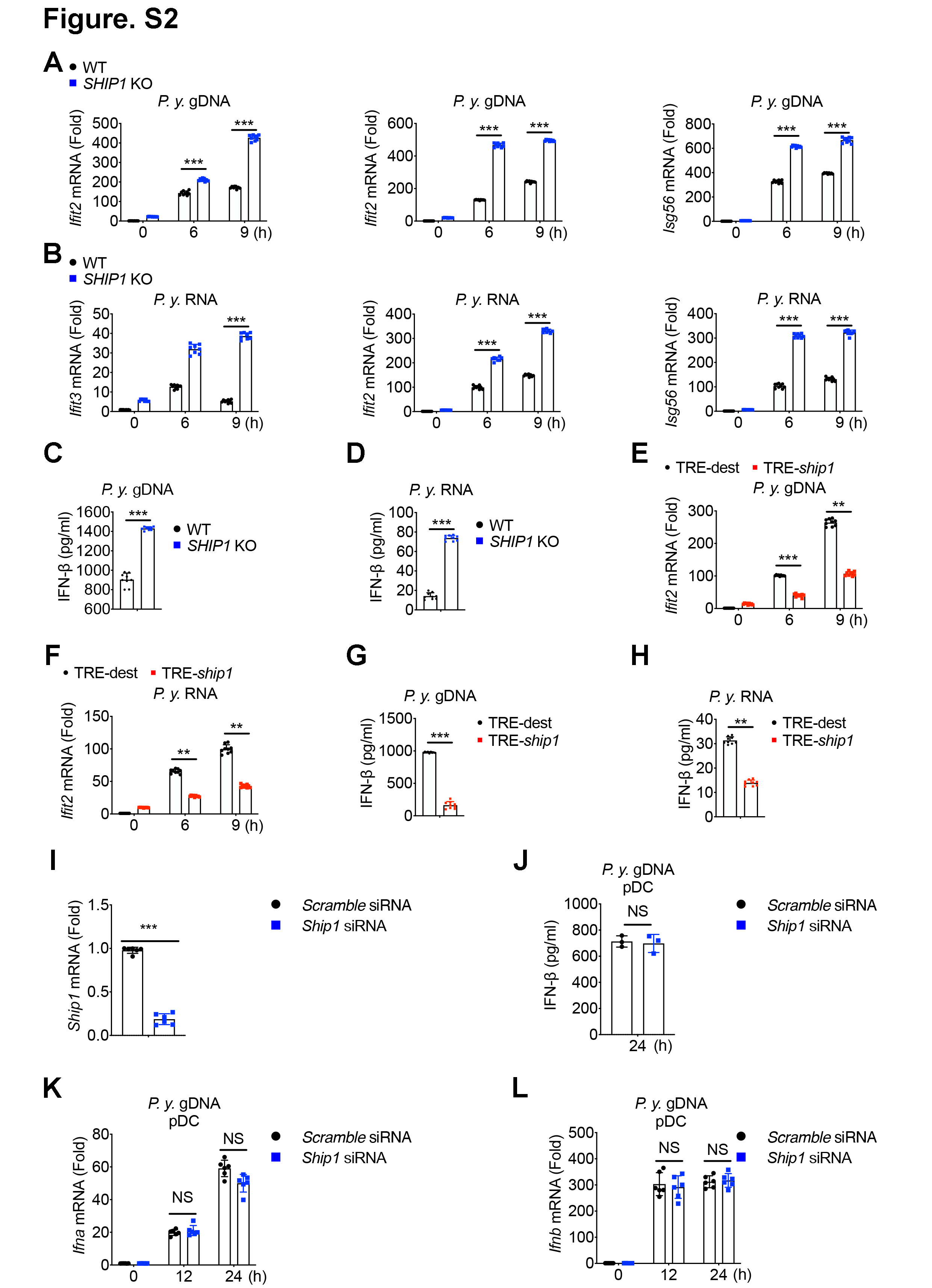

Supplement: Fig S2 — SHIP1 is a negative regulator of type I IFN signaling in host antimalarial immunity. [file mbio.03512-22-s0002.tif]

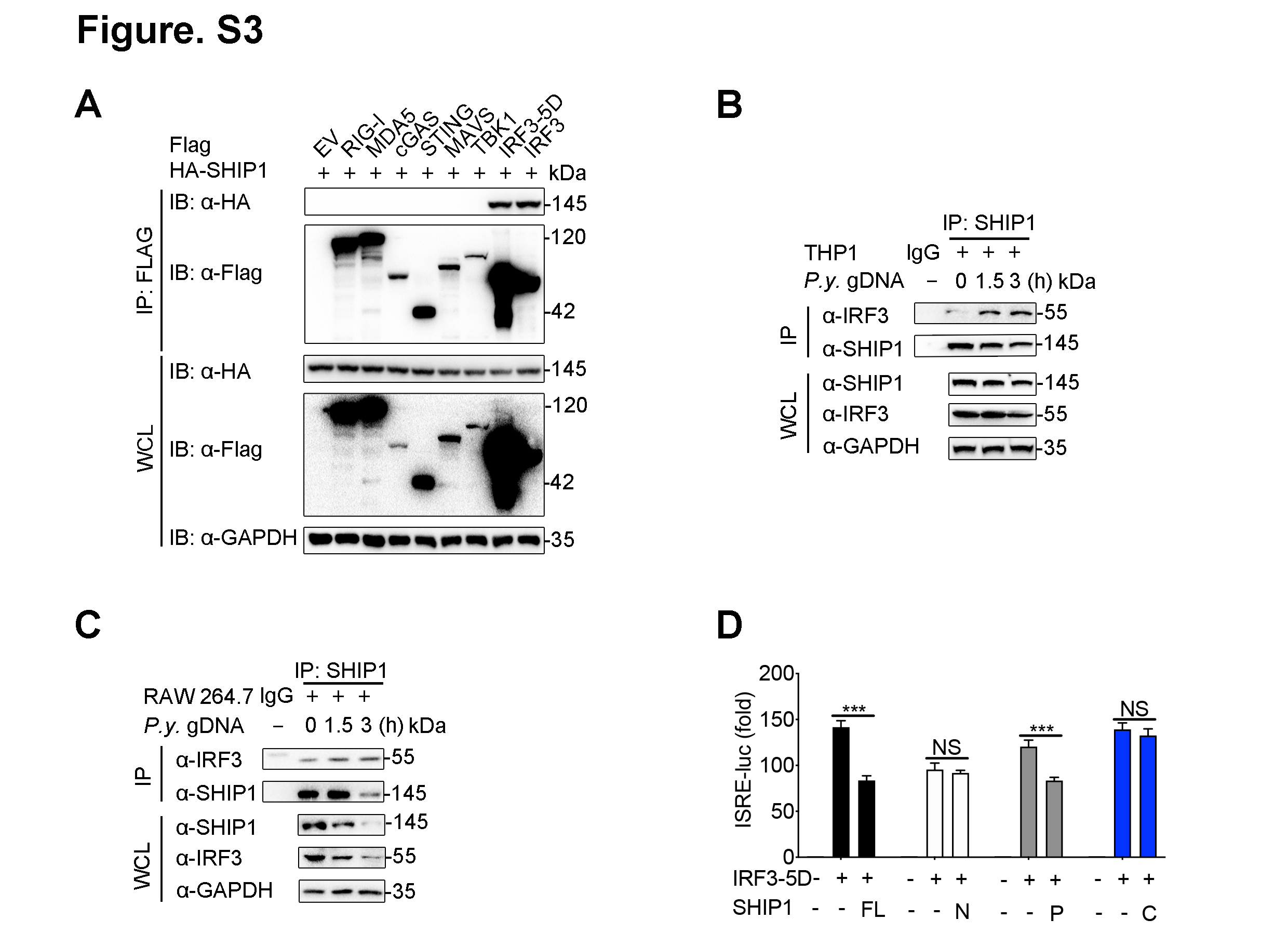

Supplement: Fig S3 — SHIP1 interacts with IRF3, but not with other molecules in type I IFN pathways. [file mbio.03512-22-s0003.tif]

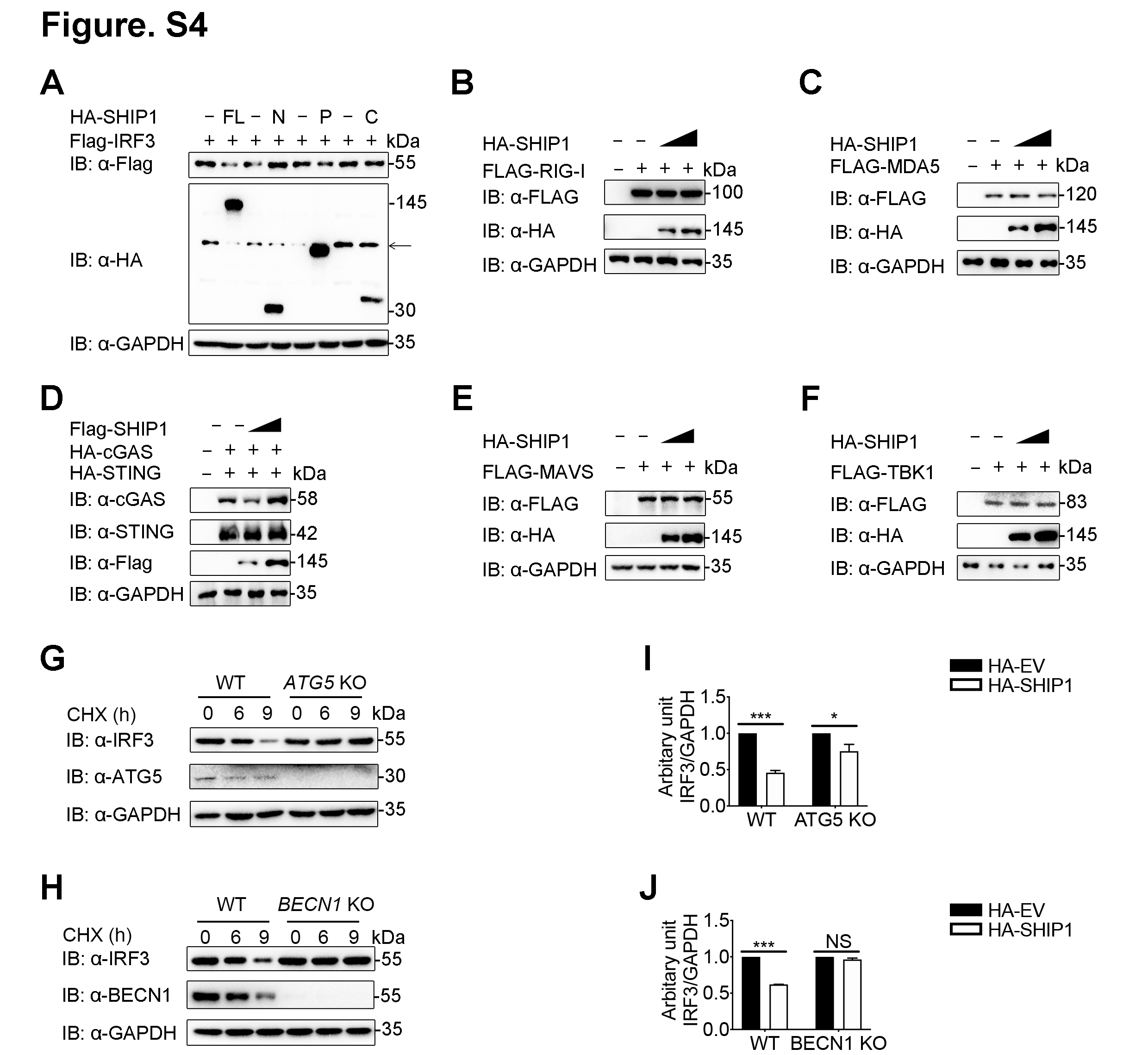

Supplement: Fig S4 — SHIP1 mediates autophagic degradation of IRF3. [file mbio.03512-22-s0004.tif]

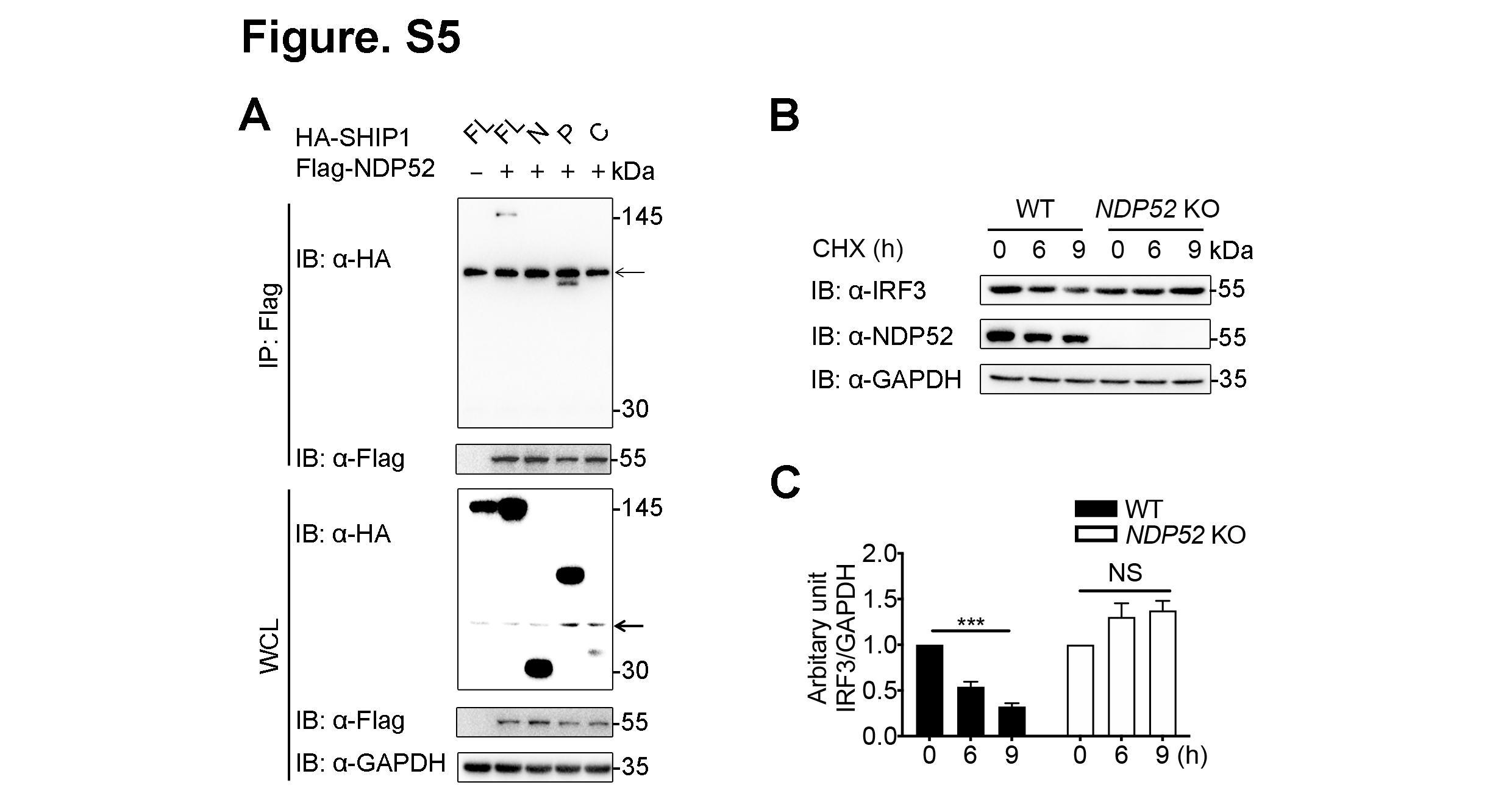

Supplement: Fig S5 — SHIP1 promotes IRF3 interacts with NDP52. [file mbio.03512-22-s0005.tif]

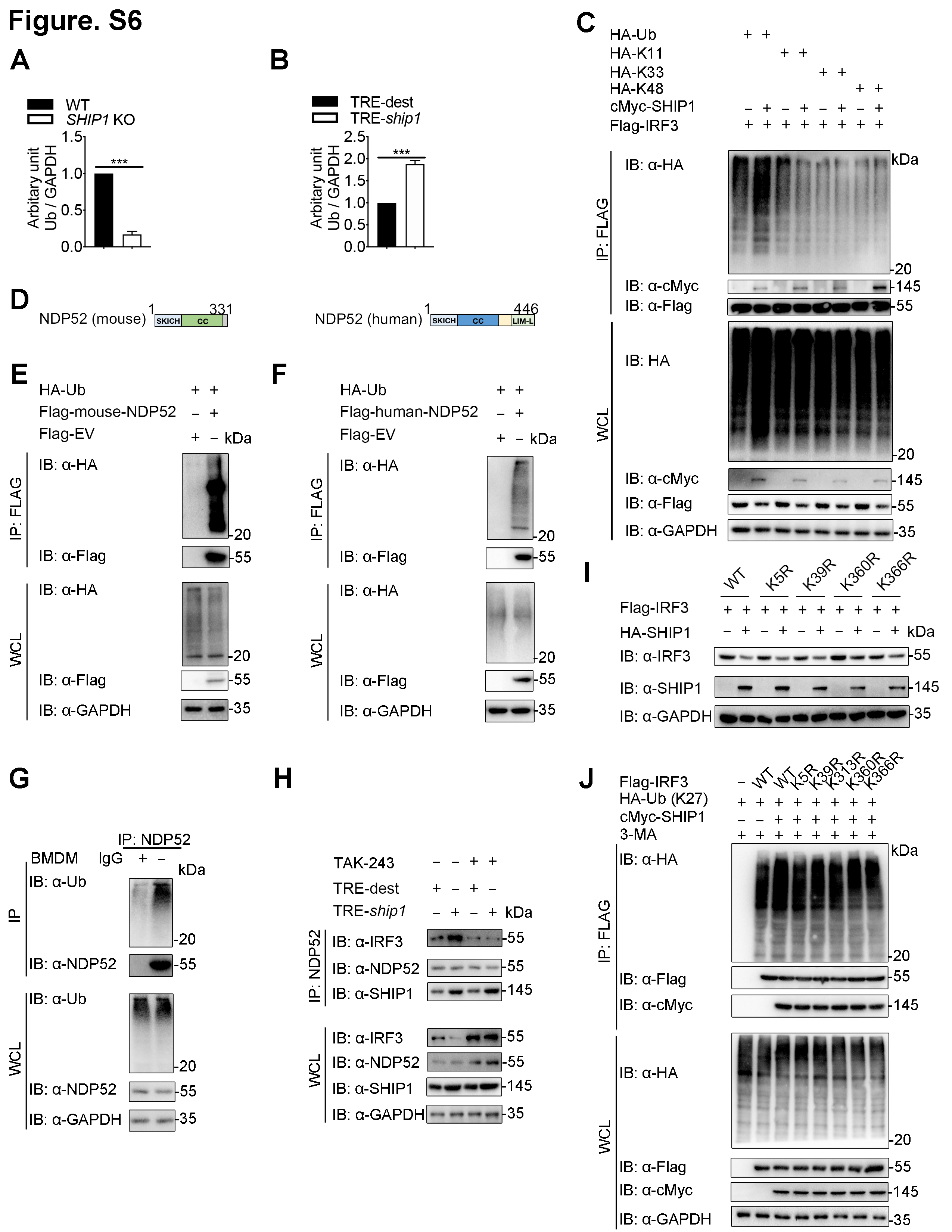

Supplement: Fig S6 — SHIP1 promotes the K63-linked ubiquitination of IRF3. [file mbio.03512-22-s0006.tif]

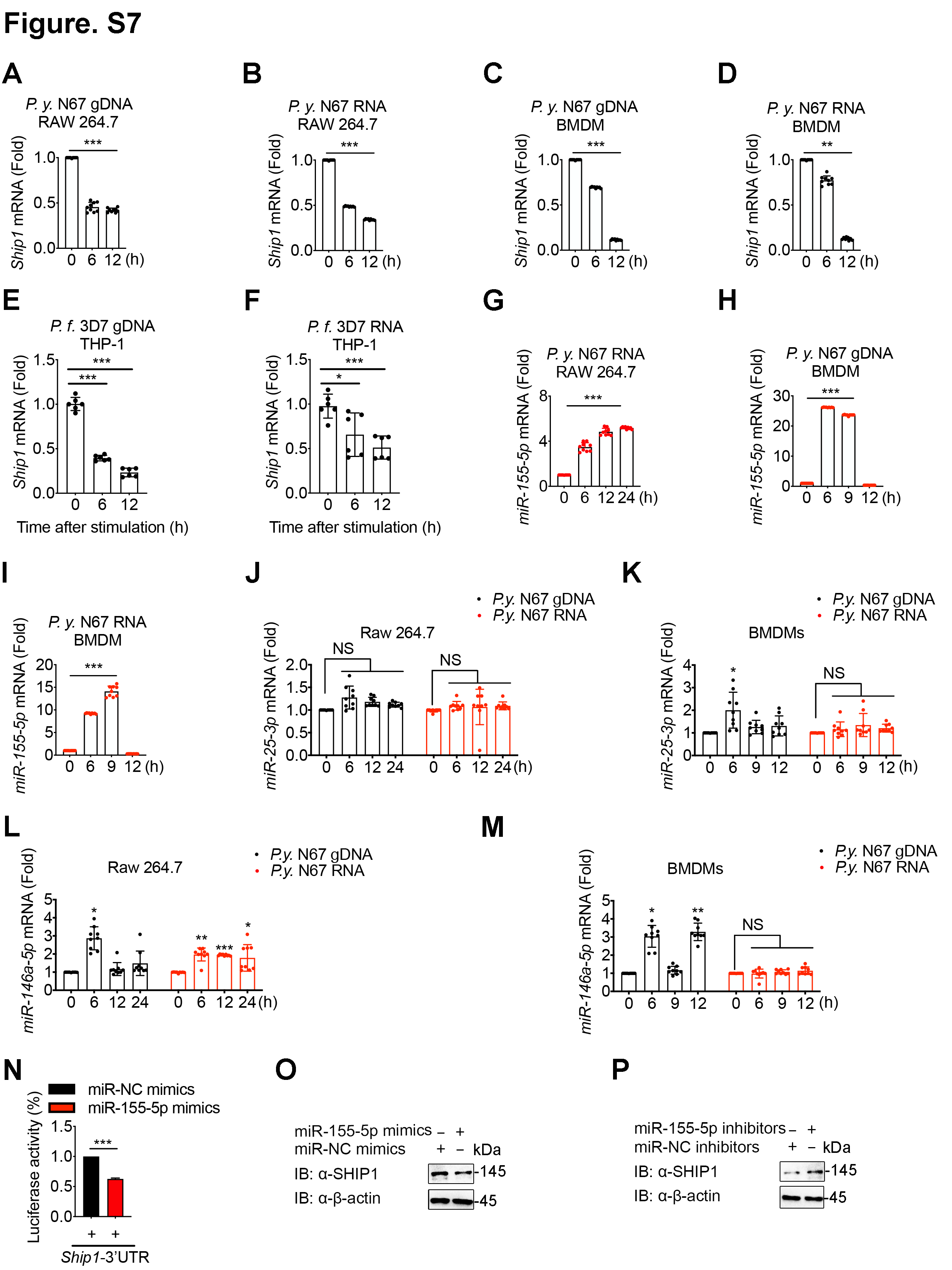

Supplement: Fig S7 — Malaria infection induces the degradation of Ship1 by upregulating miR-155-5p. [file mbio.03512-22-s0007.tif]
