# Supplementary material for: SHIP1 modulates antimalarial immunity by bridging the crosstalk between type I IFN signaling and autophagy
Source: mBio. 2023 Jun 27;14(4):e03512-22. doi: 10.1128/mbio.03512-22 (PMC10470592; doi:10.1128/mbio.03512-22)
Supplement: Legends and additional experimental details — The legends of supplemental figures; supplemental materials and methods. [file mbio.03512-22-s0008.docx]

**Supplementary Materials for**

**SHIP1 modulates antimalarial immunity by bridging the crosstalk between type I IFN signaling and autophagy**

Li *et al.*

*Corresponding author. Email: xiaoyu523@smu.edu.cn or cuij5@mail.sysu.edu.cn.

**This PDF file includes:**

Supplementary figure legends, Materials and methods

**Supplementary Figure legends**

**FIG S1 SHIP1 is detrimental for antimalarial immunity.** (A to D) mRNA levels of *Ifna* (A), *Ifnb* (B), *Tfna* (C), and *Il6* (D) in the bone marrow of WT and *Ship*-chimeric mice (n=9) on day 1 and 6 after *P.y.* N67 infection. (E and F) mRNA levels of *Ifna* (E) and *Ifnb* (F) in the bone marrow of WT and *Ship1*-chimeric mice (n=4) at 24 h after *P.b.* ANKA infection. (G) Dynamics of parasitemia after infection of WT and *Ship1*-chimeric mice (n=4) with *P.y.* 17XNL iRBCs (*i.p.,* 1×10^5^). Data in (A to G) are means ± SEM of at least three independent experiments, *^**^P*<0.01, *^***^P*<0.001; NS, not significant (two-tailed Student’s *t* test).

**FIG S2 SHIP1 is a negative regulator of type I IFN signaling in host antimalarial immunity.** (A and B) mRNA levels for *Ifit3, Ifit2,* and *Isg56* in WT and *SHIP1 KO* RAW 264.7 cells at 0, 6, and 9 h after *P.y.* gDNA (1 μg) (A) or RNA (1 μg) (B) stimulation. (C and D) The protein levels of IFN-β in WT and *SHIP1* KO RAW 264.7 cells at 24 h after *P.y.* gDNA (1 μg) (C) or RNA (1 μg) (D) stimulation. (E and F) mRNA levels for *Ifit2* in WT and *Ship1* doxy-inducible RAW 264.7 cells at 0, 6, and 9 h after *P.y.* gDNA (1 μg) (E) or RNA (1 μg) (F) stimulation. (G and H) The protein levels of IFN-β in WT and *Ship1* doxy-inducible RAW 264.7 cells at 24 h after *P.y.* gDNA (1 μg) (G) or RNA (1 μg) (H) stimulation. (I) The efficiency of *Ship1* siRNA in pDCs, mRNA level of *Ship1* at 24 h after siRNA transfection. (J) Protein levels of IFN-β (J) in the supernatant of *scramble* siRNA and *Ship1* siRNA -pDCs at 24 h after *P.y.* gDNA (1 μg) stimulation. (K and L) mRNA levels for *Ifna* (K) */ Ifnb* (L) in *scramble* siRNA and *Ship1* siRNA-pDCs at 0, 12, and 24 h after *P.y.* gDNA (1 μg) stimulation. Data in (A to L) are means ± SEM of at least three independent experiments, *^**^P*<0.01, *^***^P*<0.001; NS, not significant (two-tailed Student’s *t* test).

**FIG S3 SHIP1 interacts with IRF3, but not with other molecules in type I IFN pathways.** (A) Co-IP and immunoblot analysis of 293T cells transfected with HA-SHIP1 together with Flag-tagged RIG-I, MDA5, cGAS, STING, MAVS, TBK1, IRF3-5D or IRF3 were shown. (B) Extracts of THP-1 cells stimulated with *P.y.* gDNA (1 μg) for various times were subjected to IP with anti-SHIP1 and immunoblot analysis with indicated antibodies. (C) Extracts of RAW 264.7 cells stimulated with *P.y.* gDNA (1 μg) for various times were subjected to IP and immunoblot analyses with indicated antibodies. (D) 293T cells were transfected with the ISRE luciferase reporter, and Flag-IRF3-5D along with or without HA-SHIP1 or its indicated mutants. Data in (D) are means ± SEM of at least three independent experiments, *^***^P*<0.001; NS, not significant (two-tailed Student’s *t* test).

**FIG S4 SHIP1 mediates autophagic degradation of IRF3.** (A) 293T cells were transfected with plasmids encoding Flag-IRF3 and HA-SHIP1 or its indicated domains. The cell lysates were analyzed by immunoblot (Arrow denotes nonspecific bands). (B to F) Immunoblot analysis of extracts of 293T cells transfected with expression vectors for Flag-RIG-I, MDA5, cGAS, STING, MAVS, TBK1, and increasing vector for HA-SHIP1. (G and H) WT and *ATG5* KO (*G*) or *BECN1* KO (*H*) 293T cells were treated with cycloheximide (CHX, 100 μg/ml) for indicated times, and the cell lysates were analyzed by immunoblot. (I and J) Plots of scanned signals from (I) and (J) of main figure 4, respectively. Data in (I and J) are means ± SEM of at least three independent experiments, *^*^P*<0.05*, ^***^P*<0.001; NS, not significant (two-tailed Student’s *t* test).

**FIG S5 SHIP1 promotes IRF3 interacts with NDP52.** (A) Co-IP and immunoblot analysis of 293T cells transfected with plasmids encoding Flag-NDP52 and HA-SHIP1 or its indicated domains (Arrow denotes nonspecific bands). (B) WT and *NDP52* KO 293T cells were treated with cycloheximide (CHX,100 μg/ml) for the indicated times, and the cell lysates were analyzed by immunoblot. (C) Plots of scanned signals from (B). Data in (C) are means ± SEM of at least three independent experiments, *^***^P*<0.001; NS, not significant (two-tailed Student’s *t* test).

**FIG S6 SHIP1 promotes the K63-linked ubiquitination of IRF3.** (A and B) Plots of scanned signals from main figure 6 (A) and (B). (C) Lysates of 293T cells transfected with plasmids expressing Flag-IRF3 and HA-tagged ubiquitin (Ub) or its indicated mutants, and the empty vector or expression vector of cMyc-SHIP1. The cells were treated with 3-MA (10 mM) and the cell lysates were immunoprecipitated with anti-Flag beads and immunoblotted with anti-HA antibody. (D) The structure of NDP52 (human and mouse). (E and F) Lysates of 293T cells transfected with plasmids expressing Flag-mouse-NDP52/ Flag-human-NDP52 and HA-tagged ubiquitin (Ub). (G) Extracts of BMDMs were subjected to IP with anti-NDP52 and immunoblot analysis with indicated antibodies. (H) Co-IP and immunoblot analysis of WT and Flag-SHIP1 doxy-inducible RAW 264.7 cells. After 4 h of TAK-243 (1 μM) treatment, protein extracts were immunoprecipitated using anti-NDP52 antibody and analyzed on immunoblot using indicated antibodies. (I) 293T cells were transfected with plasmids encoding HA-SHIP1 and Flag-IRF3 or its indicated mutants, the lysates were analyzed by immunoblot after 24 h of transfection. (J) 293T cells transfected with plasmids expressing HA-K27-linked-Ub, Flag-IRF3 or its indicated mutants, together with the empty vector or expression vector of c-Myc-SHIP1, were treated with 3-MA (10 mM). The cell lysates were immunoprecipitated with anti-Flag beads and immunoblotted with anti-HA. Data in (A and B) are means ± SEM of at least three independent experiments, *^***^P*<0.001; (two-tailed Student’s *t* test).

**FIG S7 Malaria infection induces the degradation of *Ship1* by upregulating miR-155-5p.** (A to D) mRNA levels of *Ship1* in RAW 264.7 cells (A and B) or BMDMs (C and D) were detected using qPCR after *P.y.* gDNA (1 μg) or RNA (1 μg) stimulation for the indicated times. (E and F) mRNA levels of *Ship1* in THP-1 cells were detected using qPCR after *P.f.* 3D7 gDNA (E) or RNA (F) stimulation for the indicated times. (G to I) mRNA levels of miR-155-5p were detected in RAW 264.7 cells (G) or BMDMs (H and I) after *P.y.* gDNA or RNA stimulation for the indicated times. (J and K) mRNA levels of miR-25-3p were detected in RAW 264.7 cells (J) or BMDMs (K) after *P.y.* gDNA or RNA stimulation for the indicated times. (L and M) mRNA levels of miR-146a-5p were detected in RAW 264.7 cells (L) or BMDMs (M) after *P.y.* gDNA or RNA stimulation for the indicated times. (N) 293T cells were transfected with plasmid encoding psi-check2-*Ship1*-3’UTR (untranslated region) and miR-NC or miR-155-5p mimics. Luciferase activities were measured and plotted. (O and P) RAW 264.7 cells were treated with mimics (O) or inhibitors (P) of miR-NC or miR-155-5p, and protein levels of SHIP1 were detected by immunoblot. Data in (A to N) are means ± SEM of at least three independent experiments, *^*^P*<0.05*, ^**^P*<0.01, *^***^P*<0.001; NS, not significant (two-tailed Student’s *t* test).

**Materials and Methods**

**Immunofluorescence assay**

The immunofluorescence assays and confocal microscopy were conducted as described previously. BMDMs grown on glass-bottom dish (MatTek) in complete medium, with *scramble* SIRNA or *Ship1* SIRNA for 24 h, and infected with *P.y* gDNA for indicated time; *SHIP1* KO RAW 264.7 cells, Flag-SHIP1 inducible RAW 264.7 cells grown on glass-bottom dish (MatTek) in complete medium. Cells were then fixed in 4% paraformaldehyde solution in PBS at room temperature for 15 min and permeabilized at room temperature with 0.1% Triton X-100/PBS. Specific antibodies for detecting endogenous SHIP1, IRF3 and NDP52 were used in 1:200 dilution. Cells were then washed three times with PBS and incubated with secondary fluorescent antibody (1:500 dilution, antibodies are listed in the Key Resources Table) for 1 h. The nucleus was then labeled with DAPI for 5 min in the dark and then followed by three washes in PBS. Samples were then visualized using Nikon Eclipse Ti-E microscope. All acquired images were analyzed and the correlation coefficient (r) of pixel intensity values was extracted by using the Nikon NIS-Elements AR package or the ImageJ (NIH) software.

**Plasmids**

HA- or Flag-tagged SHIP1, RIG-I, MDA5, cGAS, STING, MAVS, TBK1, IRF3, and NDP52 were constructed using Vazyme ClonExpress MultiS One Step Cloning Kit (C113-01/02). TIANGEN Fast Site-Directed Mutagenesis Kit (KM101) was used to construct mutant plasmids.

**Culture of *P.f.* 3D7**

*P.f.* 3D7 was cultured in fresh human red blood cells at 37 °C, 5% CO_2_, 5% O_2_ in RPMI-1640 medium containing 5 g/l Albumax. Parasites were synchronized with Percoll. Fifteen hours after Percoll, the parasites had developed to the ring stage with around 5% parasitemia.

**Luciferase and reporter assays**

HEK293T (0.5×10^6^) cells were plated in 24-well plates and transfected various plasmids using Genstar high-efficiency transfect reagent (C101-10). Plasmids used include those encoding IFN-β or ISRE luciferase reporter (firefly luciferase; 200 ng), pRI-TK (renilla luciferase plasmid; 50 ng), 400 ng of Flag-RIG-I, Flag-cGAS, Flag-STING, Flag-MDA5, Flag-MAVS, Flag-TBK1 or Flag-IRF3 (5D), and increasing concentrations (0, 300, 600 ng) of plasmids expressing SHIP1. Empty pcDNA3.1 vector was used to maintain equal amounts of DNA among wells. Cells were collected at 24 h after transfection and luciferase activity was measured using the Dual-Luciferase Assay (Promega) with a Luminoskan Ascent luminometer (Thermo Scientific), according to the manufacturer’s protocol. Reporter gene activity was determined by normalization of the firefly luciferase activity to renilla luciferase activity.

**Immunoprecipitation and immunoblot analysis**

For immunoprecipitation, whole cell extracts were prepared after transfection or stimulation with appropriate ligands, followed by incubation overnight with the appropriate antibodies plus anti-Flag beads (Sigma) or Protein A/G beads (Pierce). Beads were washed with low-salt lysis buffer, and immunoprecipitates were eluted with 2×SDS Loading Buffer (Cell Signaling Technology) and resolved on SDS-PAGE. Proteins were transferred to PVDF membranes (Bio-Rad) and further incubated with the appropriate antibodies. LumiGlo Chemiluminescent Substrate System (KPL) was used for protein detection.

**Antibodies**

The following antibodies were used in this study: anti-SHIP1 (sc-8425), Phospho-TBK1/NAK (Ser172) Rabbit mAb (CST-5483S), TBK1/NAK Antibody (CST-3013S), Phospho-IRF-3 (Ser396) Rabbit mAb (CST-4947S), IRF3 (proteintech-11312-1-AP), Anti-HA-Peroxidase (sigma-12013819001), Anti-FLAG M2-Peroxidase Ab (sigma-A8592), ATG5 (proteintech-10181-2-AP), BECN1 (proteintech-11306-1-AP), NDP52 (proteintech-12229-1-AP), p62/SQSTM1 (proteintech-18420-1-AP), anti-c-Myc-Peroxidase (sigma-11814150001), anti-Ubiquitin (sc-8017), GAPDH (RM2002), β-actin Mouse Monoclonal Antibody (RM2001).

**RNA Extraction and Quantitative RT-PCR**

Total RNA was extracted using TRIzol reagent (Invitrogen) and reverse-transcribed using oligo-dT primers and reverse transcriptase (Genstar). Real-time quantitative PCR was performed using SYBR green qPCR Mix kit (Genstar) and specific primers designed using the Primer 5.0 analyzer (Applied Biosystems). Data were normalized to the GADPH gene, and the relative abundance of transcripts was calculated by the 2^-∆∆Ct^ models. The following primers were used for real-time PCR:

| **Description gene** | **Forward Primer Sequence** | **Reverse Primer Sequence** |
| --- | --- | --- |
| mouse *Ifna* | GGACTTTGGATTCCCGCAGGAGAAG | GCTGCATCAGACAGCCTTGCAGGTC |
| mouse *Ifnb* | TCACCTACAGGGCGGACTTC | TCTCTGCTCGGACCACCATC |
| mouse *Tnfa* | GACGTGGAACTGGCAGAAGAG | TTGGTGGTTTGTGAGTGTGAG |
| mouse *Il6* | CTCTGGGAAATCGTGGAAAT | CCAGTTTGGTAGCATCCATC |
| mouse *Ship1* | GCCCCTGCATGGGAAATCAA | TGGGTAGCTGGTCATAACTCC |
| mouse *Isg56* | TGCGATCCACAGTGAACAAC | ACTTCCGGGAAATCGATGAG |
| mouse *Ifit3* | CCTACATAAAGCACCTAGATGGC | ATGTGATAGTAGATCCAGGCGT |
| mouse *Ifit2* | GGAGAGCAATCTGCGACAG | GCTGCCTCATTTAGACCTCTG |
| mouse *Gapdh* | AAGGTCATCCCAGAGCTGAA | CTGCTTCACCACCTTCTTGA |
| mouse *miR-25-3p* | GCCATTGCACTTGTCTCG | GCAGGGTCCGAGGTATTC |
| mouse *miR-146a-5p* | CGTGTGAGAACTGAATTCCA | GCAGGGTCCGAGGTATTC |
| mouse miR-155-5p | CCGGTTAATGCTAATTGTGAT | GCAGGGTCCGAGGTATTC |
